# Context-Guided Hard-Negative Background Suppression for Crack Segmentation on Complex-Texture Farmland Roads

**DOI:** 10.3390/s26165185

**Published:** 2026-08-16

**Authors:** Niangzhi Mao, Shihai Ding, Yajie Zhang, Xiaoping Chen, Changfa Ai, Bowen Zhou

**Affiliations:** 1 School of Architecture and Civil Engineering, Chengdu University, Chengdu 610106, China; 18381019681@163.com (N.M.);; 2 College of Civil Engineering, Southwest Jiaotong University, Chengdu 610031, China

**Keywords:** field roads in high-standard farmland, crack segmentation, semantic segmentation, skip connections, hard-negative mining, background texture suppression

## Abstract

Field-road cracks in high-standard farmland are often slender, low-contrast, and surrounded by complex textures that cause U-Net-based models to misclassify aggregates, tire marks, shadows, and repair edges as cracks. To reduce these false positives, this study develops a task-oriented context-guided hard-negative background suppression network (CGHN-Net) based on Squeeze-and-Excitation U-Net (SE-U-Net). The context-guided skip gate (CGSG), a same-resolution adaptation of additive attention gating, uses already upsampled decoder features as semantic guides to filter encoder skip features at all three scales. Hard-negative background suppression loss (HNBS Loss), a background-restricted hard-example mining objective, further targets elevated-probability responses within ground-truth background regions. The dataset comprised 2235 vehicle-acquired grayscale pavement images from independent sessions and mutually exclusive road sections: 1684 for training, 464 for validation, and 87 for testing. Across three random seeds, CGHN-Net achieved Dice, IoU, precision, recall, and FP area ratio values of 0.8682 ± 0.0055, 0.7791 ± 0.0102, 0.8780 ± 0.0161, 0.8734 ± 0.0266, and 0.0042 ± 0.0008, respectively. Against U-Net, Attention U-Net, UNet++, DeepLabV3+, SegFormer-B0, and BGCrack, it achieved the highest Dice, IoU, and recall, indicating the strongest overall overlap and crack recovery. Sequence-aware paired analysis against UNet++ preserved contiguous acquisition order through block lengths of 3, 5, and 10 images, and all block-bootstrap confidence intervals excluded zero. On 100 held-out crack-free images, CGHN-Net also achieved the lowest post-processed image-level false-alarm rate and FP area ratio among the included models. Additional three-seed validation on the independently acquired public CrackForest Dataset (CFD), with the selected models retrained on mutually exclusive CFD partitions, showed that CGHN-Net achieved Dice, IoU, and recall of 0.6679 ± 0.0162, 0.5028 ± 0.0182, and 0.9486 ± 0.0085, respectively, exceeding UNet++ and BGCrack in overlap and crack recovery. The results support the task-oriented combination of same-resolution skip filtering and background-restricted hard-example mining for suppressing texture-induced false responses, while the CFD experiment is interpreted as independent public-dataset retraining rather than zero-shot transfer.

## 1. Introduction

Pavement cracking is one of the most common forms of distress that develop during road service. If cracks are not accurately identified and treated at an early stage, water may infiltrate the pavement surface and base layers through the crack openings, thereby accelerating the development of potholes, settlement, alligator cracking, and other structural distresses. These defects can substantially increase subsequent maintenance costs and compromise traffic safety. For field roads in high-standard farmland, the pavement serves essential functions such as agricultural machinery access, transportation of agricultural supplies, and transfer of agricultural products. Its service condition is therefore closely related to agricultural production efficiency and infrastructure management. Accordingly, the development of an efficient and robust automatic crack segmentation method capable of adapting to complex pavement textures is of considerable engineering significance.

Traditional crack detection primarily relies on manual inspection and image-processing techniques such as threshold segmentation, edge detection, and morphological operations. Manual inspection is labor-intensive, inefficient, and highly subjective, whereas methods based on edge information, grayscale thresholds, and morphological rules are sensitive to shadows, oil stains, tire marks, coarse aggregates, and repair edges [1,2,3]. With the rapid development of convolutional neural networks and semantic segmentation, fully convolutional networks (FCNs) extended conventional classification networks to end-to-end pixel-wise prediction [4]. U-Net integrates shallow spatial details with deep semantic information through an encoder–decoder architecture and skip connections [5]. SegNet, PSPNet, and DeepLabV3+ further improve pixel-level segmentation performance from the perspectives of decoder-based feature reconstruction, global context aggregation, and atrous convolution, respectively [6,7,8]. Attention U-Net and UNet++ subsequently enhance multiscale feature fusion through attention gating and nested skip connections [9,10], providing important foundations for the segmentation of small targets and objects with irregular boundaries. More recently, SegFormer combines a hierarchical Transformer encoder with a lightweight multilayer perceptron decoder to provide an efficient modern semantic segmentation framework [11].

To address the slender morphology, low contrast, and complex backgrounds of pavement cracks, numerous studies have adapted general-purpose segmentation networks for crack-specific applications. The CrackNet series performed pixel-level crack detection using vehicle-mounted pavement imagery and promoted the application of deep neural networks to pavement crack recognition [12,13,14]. Yang et al. employed fully convolutional networks for crack detection and measurement [15], while Ai et al. enhanced pixel-level crack representation by incorporating multiscale neighborhood information [16]. Mei et al. further investigated a cost-effective crack inspection framework combining low-cost cameras with deep neural networks [17]. Meanwhile, attention mechanisms such as squeeze-and-excitation (SE) and the convolutional block attention module (CBAM) enhance informative features through channel-wise or spatial recalibration [18,19]. For complex road backgrounds, DCM-Net introduces attention mechanisms into skip connections to alleviate interference from lane markings, manholes, and background noise [20], whereas improved U-Net-based methods employ strip pooling and attention modules to strengthen the representation of slender cracks [21]. BGCrack further introduces explicit boundary-feature modeling, global context modeling, and joint crack-boundary supervision for crack segmentation on steel structures [22]. These studies indicate that, although skip connections are beneficial for recovering fine crack details, insufficient feature filtering may also allow low-level texture noise to be propagated into the decoder.

In addition to feature fusion, the proportion of crack pixels is substantially lower than that of background pixels, making class imbalance and hard-example mining important factors that affect model performance. Detection frameworks such as YOLO, Faster R-CNN, EfficientDet, and YOLOv4 have promoted research on multiscale feature fusion and hard-example mining strategies for road distress recognition [23,24,25,26]. Recent studies based on improved YOLOv8s further indicate that attention mechanisms, feature fusion strategies, and loss-function design can improve crack recognition under complex conditions [27]. In pixel-level segmentation, Dice loss directly optimizes the overlap between predicted and ground-truth regions [28]. Focal loss down-weights easy samples while retaining contributions from all pixels [29]. Online hard-example mining (OHEM) explicitly selects high-loss examples during training [30], whereas bootstrapped cross-entropy retains a fixed number of difficult pixels in semantic segmentation [31]. Tversky loss balances precision and recall by adjusting the penalties assigned to false-positive and false-negative predictions [32]. Studies on GIoU and DIoU also demonstrate, from the perspective of task-oriented error constraints, that appropriately designed optimization objectives can improve hard-example mining [33,34]. However, the dominant errors observed in the field-road images considered in this study are not caused solely by the sparsity of crack pixels. A small number of coarse particles, dark spots, shadow boundaries, and repair textures are also assigned relatively high crack probabilities and are consequently misclassified as genuine cracks. Motivated by the problem of elevated-probability spurious crack responses induced by complex pavement textures, this study proposes CGHN-Net. At the feature-fusion level, context-guided skip filtering is employed to suppress low-level texture interference; at the optimization level, hard-negative background constraints are introduced to further reduce elevated-probability false-positive responses in background regions.

The task-oriented methodological contributions and empirical findings of this study are summarized as follows: (1) CGSG is incorporated at all three skip-connection scales of the SE-U-Net backbone. It follows the general formulation of additive attention gating but uses the already upsampled decoder feature as a same-resolution semantic guide, avoiding internal feature resampling before skip fusion. Its contribution is an application-specific, full-scale deployment for texture-oriented filtering rather than a new attention principle. (2) HNBS Loss is formulated as a background-restricted hard-pixel mining objective. It follows established loss-based hard-example selection but restricts the candidate set to ground-truth background pixels with elevated predicted crack probabilities, targeting spurious crack responses from coarse textures, shadows, and repair edges. (3) The method is evaluated on vehicle-acquired field-road images using three random seeds, sequence-aware paired statistics, a held-out crack-free subset, controlled comparisons, ablations, efficiency measurements, and an additional public CFD experiment. The CFD models were retrained on an independently acquired road and sensor domain, providing independent public-dataset validation after retraining without being interpreted as zero-shot cross-domain transfer. These experiments assess the practical value of the two task adaptations without claiming a fundamentally new attention or hard-mining principle.

## 2. Sensing System and Image Acquisition

### 2.1. Vehicle-Mounted Sensing System

The images were acquired on high-standard farmland roads in Huzhou, China, using a multifunctional inspection system mounted on a modified Ford Transit. The platform integrated an industrial CCD camera, a laser-triangulation scanner, GPS, and an IMU, with an approximate pavement measurement resolution of 1 mm. Table 1 summarizes the system configuration.

### 2.2. Image Acquisition Procedure

The vehicle operated at approximately 5–15 km/h in crack-dense areas and 15–30 km/h on regular sections, with sampling intervals of about 10 and 20 cm, respectively. A polarizing filter reduced glare, dynamic exposure adapted to illumination, and vibration isolation limited motion-induced instability.

**Figure 1:**
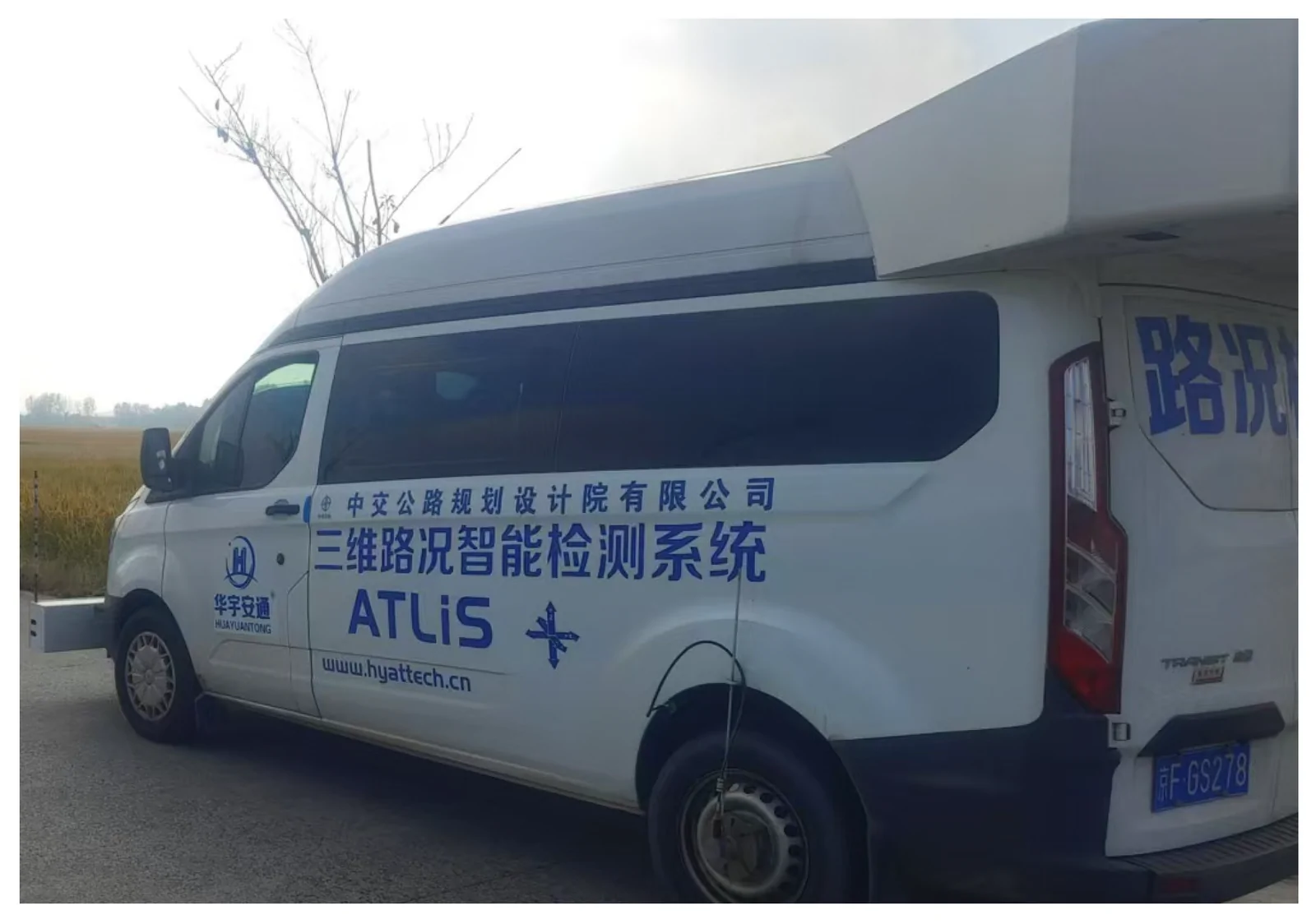
Multifunctional pavement inspection vehicle.

Figure 1 shows the acquisition vehicle. Images were checked during and after collection; frames with more than 30% occlusion by vehicles, pedestrians, vegetation, or other objects were excluded or reacquired. Retained images preserved realistic interference from aggregates, dark spots, shadows, tire marks, voids, contamination, and repair textures.

### 2.3. Acquisition Sessions and Dataset Partition

Data were collected during three independent sessions on different days and on mutually exclusive road sections. Training images came from Road Section A on day 1, validation images from Sections B and C on day 2, and test images from Road Section D on day 3. Each road section and continuous sequence belonged to only one partition. Within each sequence, image identifiers preserve the original acquisition order. Because the 87 test images were acquired consecutively at short spatial intervals, neighboring test images may exhibit local spatial and sequential correlation; this dependence is addressed in the sequence-aware paired statistical analysis described in Section 4.3.

The dataset contained 2235 images: 1684 for training, 464 for validation, and 87 for testing. Among the 464 validation images, 168 contained annotated cracks, and 296 were crack-free. The complete validation set was used for routine model validation and checkpoint selection, while the HNBS parameter-sensitivity analysis used only the 168 crack-containing validation images, as specified in Section 3.5.2. The test set was reserved for final evaluation.

A further 100 unused crack-free images from Road Section D on day 3 formed a held-out negative subset. They were excluded from all training, validation, and crack-containing test data and contained difficult textures but no visible cracks, as confirmed by manual review before model inference. Because they came from the same session as the test set, they provide an in-distribution false-positive evaluation rather than a cross-road or cross-domain test.

Exact duplicates were screened with SHA-256 hashes, and potential cross-partition near-duplicates were identified by perceptual hashing and manual review. No exact duplicates or confirmed cross-partition near-duplicate pairs were found.

## 3. Methodology and Methodological Positioning

### 3.1. Overall Architecture

**Figure 2:**
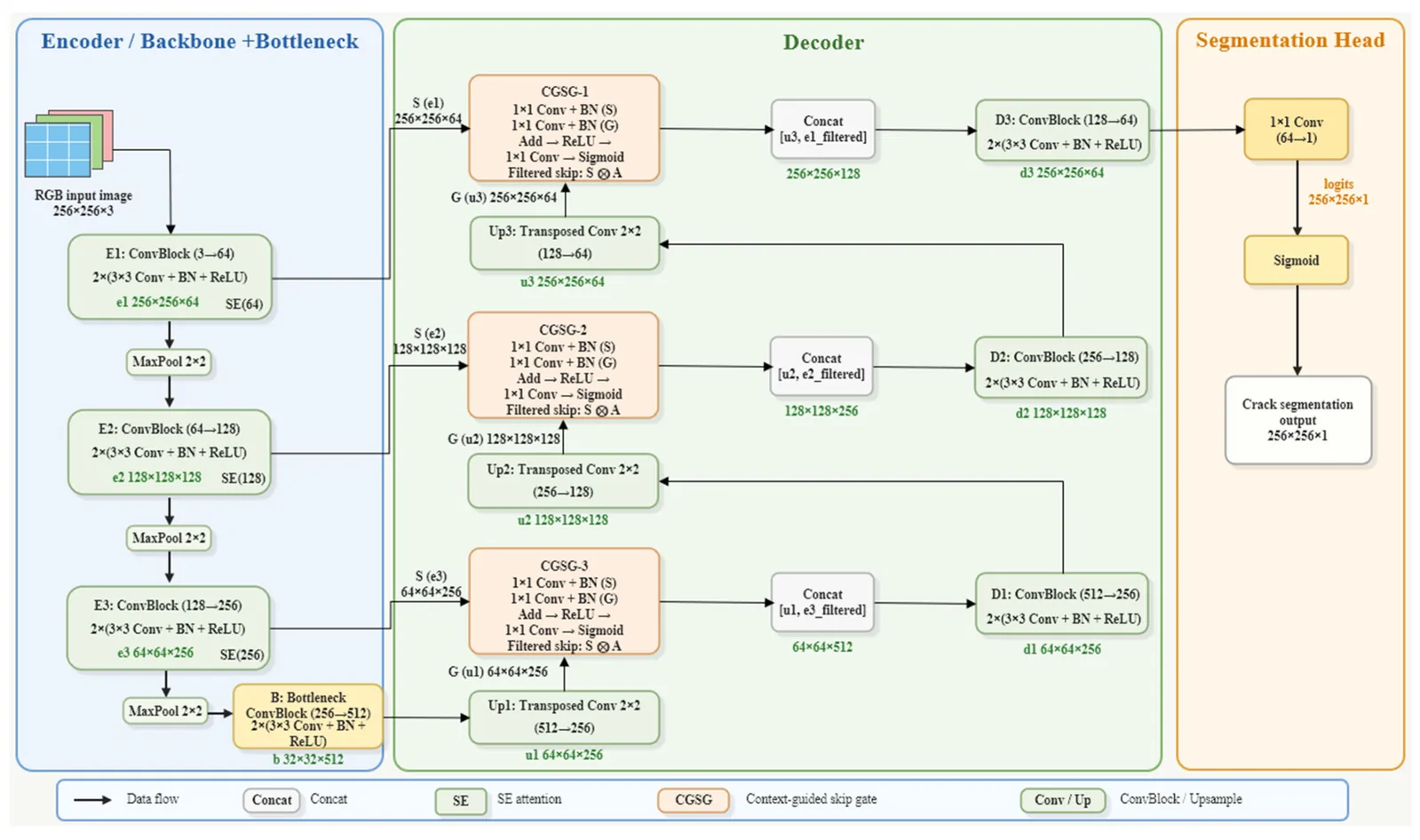
Overall architecture of CGHN-Net.

The proposed CGHN-Net is built on an SE-U-Net backbone, as shown in Figure 2. Original 512 × 512 grayscale images were resized to 256 × 256 and replicated across three channels. The 256 × 256 input was adopted because 512 × 512 training could not be applied consistently to all comparison models with a batch size of 8 on the available 8 GB GPU and substantially increased training time. The same input setting was used for every model. The encoder contains three stages with 64, 128, and 256 channels and a 512-channel bottleneck. Each stage uses two 3 × 3 Conv-BN-ReLU blocks followed by SE recalibration. The decoder uses three 2 × 2 transposed convolutions and fuses each upsampled feature with the corresponding CGSG-filtered skip feature. A final 1 × 1 convolution produces single-channel logits, which are converted by a sigmoid function into a 256 × 256 crack-probability map.

Figure 2 summarizes four stages: multiscale encoding, bottleneck aggregation, progressive decoding, and pixel-wise prediction. Unlike U-Net, CGHN-Net uses the upsampled decoder features u3, u2, and u1 to gate encoder features e1, e2, and e3 before fusion with D3, D2, and D1. This retains crack details while reducing transmission of texture noise. The segmentation head outputs a probability map rather than a thresholded mask.

### 3.2. Label Extraction and Probability Output

Crack regions were annotated using red traces whose brush width followed the apparent crack width. Binary masks are extracted by an RGB threshold. For pixel *i* with intensities *R*_*i*, *G*_*i*, and *B*_*i*, the label *y*_*i* is defined as follows:

(1) y_i=1,if R_i>150 and G_i<80 and B_i<80; otherwise y_i=0

The final 1 × 1 convolution produces a logit *z*_*i*, which is converted to crack probability *p*_*i* by the sigmoid function as follows:

(2) p_i=σ(z_i)=1/(1+exp(−z_i))

Continuous probabilities are used in the Dice and Tversky losses during training. At test time, a fixed threshold of 0.5 produces the binary mask as follows:

(3) $$\hat{y}\text{\_}i=1(p\text{\_}i>0.5)$$

### 3.3. Task-Adapted Context-Guided Skip Gate

U-Net skip features contain both crack details and substantial road texture. CGSG therefore uses the upsampled decoder feature *G* to guide filtering of the encoder skip feature *S*. Both are projected to a shared channel space by 1 × 1 *Conv* + *BN*:

(4) $$S^{\prime }=BN(Conv\text{\_}\{1\times 1\}(S)),\;\;G^{\prime }=BN(Conv\text{\_}\{1\times 1\}(G))$$

The projected features are added and passed through *ReLU*, 1 × 1 convolution, and sigmoid to obtain a single-channel Attention Map A as follows:

(5) $$A=\sigma (Conv\text{\_}\{1\times 1\}(ReLU(S^{\prime }+G^{\prime })))$$

A then reweights the original skip feature:

(6) $$\hat{S}=S⊗A$$

Here, ⊗ denotes element-wise multiplication. The deeper decoder feature supplies semantic guidance, while the encoder feature retains local detail. Their projected sum generates *A* ∈ [0, 1], which is broadcast across channels to suppress irrelevant locations without changing the skip-channel count. G only determines the weights; the feature forwarded for concatenation is the filtered encoder feature $\hat{S}$.

**Figure 3:**
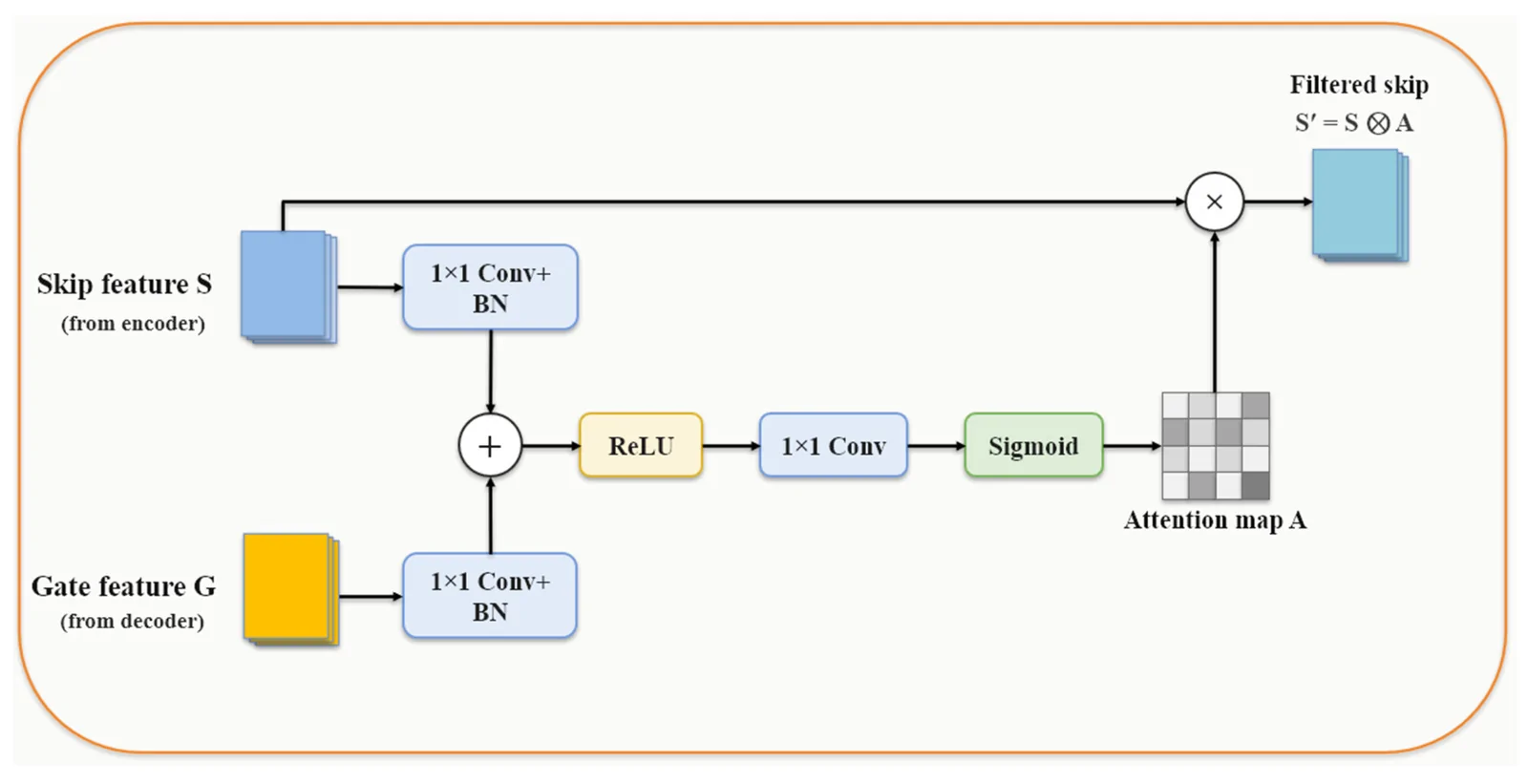
Structure of the CGSG module.

Figure 3 shows the CGSG data flow. Projected skip and gate features are added, transformed into Attention Map A, and multiplied with the original skip feature to obtain $\hat{S}$ = *S* ⊗ *A*. Decoder semantics therefore suppress texture edges inconsistent with cracks while retaining semantically consistent slender structures. The gate is applied at all three skip scales.

#### 3.3.1. Relationship to the Standard Attention Gate

CGSG follows the additive attention gate formulation used in Attention U-Net: encoder and decoder features are projected, added, and transformed into an attention map, and used to weight the encoder feature.

The difference is spatial alignment. The standard gate downsamples the encoder feature to the decoder resolution and then upsamples the attention map. CGSG instead uses the decoder feature after normal decoder upsampling, so each skip feature is filtered at its native resolution.

CGSG is applied at all three SE-U-Net skip scales. Table 2 summarizes the operation-level differences, and Section 3.3.2 reports the controlled comparison.

Table 2 shows that both gates use additive attention, while CGSG performs filtering directly at the native skip resolution.

#### 3.3.2. Controlled Comparison with the Standard Attention Gate

B0, SE-U-Net with the standard attention gate, and B1 were trained under identical conditions with HNBS Loss disabled, isolating the effect of skip gating. Table 3 and Table 4 report segmentation and efficiency results.

The standard attention gate achieved the highest precision (0.8785 ± 0.0164) and lowest FP area ratio (0.0040 ± 0.0008), but recall fell to 0.8212 ± 0.0149, yielding lower Dice (0.8403 ± 0.0058) and IoU (0.7357 ± 0.0085). It therefore suppressed background strongly but also removed weak cracks.

CGSG achieved Dice, IoU, and recall values of 0.8643 ± 0.0021, 0.7717 ± 0.0047, and 0.8769 ± 0.0172. Relative to the standard gate, these increased by 0.0240, 0.0360, and 0.0557, respectively. Although precision was 0.0115 lower and the FP area ratio 0.0007 higher, CGSG provided a better balance between false-positive suppression and crack preservation.

Compared with B0, CGSG slightly improved Dice, IoU, and precision, reduced the FP area ratio from 0.0049 to 0.0047, and decreased recall only from 0.8796 to 0.8769. Same-resolution gating therefore reduced texture interference without the pronounced weak-crack loss of the standard gate.

CGSG used 7.8121 M parameters, compared with 7.9706 M for the standard-gate variant. Its latency was 13.7077 ms/image versus 13.9419 ms/image, with FPS values of 72.95 and 71.73, respectively. Thus, CGSG achieved better segmentation performance with comparable efficiency.

### 3.4. Loss Functions

The base objective uses Dice, Focal, and Tversky losses; BCE is included as a simple reference in the controlled loss comparison. BCE is defined as:

(7) BCE_i=−[y_i log(p_i+ε)+(1−y_i)log(1−p_i+ε)]

Dice loss optimizes overlap between predicted and ground-truth crack regions:

(8) L_Dice=1−(2Σ_i p_i y_i+ε)/(Σ_i p_i+Σ_i y_i+ε)

Focal loss uses *p*_*t* = *exp*(−*BCE*_*i*), *γ* = 2, and *α* = 0.8:

(9) L_Focal=mean_i[α(1−p_{t,i})^γ BCE_i],  p_{t,i}=exp(−BCE_i)

Tversky loss balances false positives and false negatives through the following soft statistics:

(10) TP_s=Σ_i p_i y_i, FP_s=Σ_i p_i(1−y_i), FN_s=Σ_i(1−p_i)y_i

(11) L_Tversky=1−(TP_s+ε)/(TP_s+α_T FP_s+β_T FN_s+ε)

We set *α*_*T* = 0.3 and *β*_*T* = 0.7 to emphasize missed crack pixels.

### 3.5. Hard-Negative Background Suppression Loss

False positives are concentrated in a small number of crack-like background regions with high predicted probabilities. HNBS Loss targets these regions through background-restricted hard-pixel mining. For each image, the ground-truth background set is:

(12) Ω_bg={i|y_i=0}

Candidates are restricted to ground-truth background pixels with *p*_*i* ≥ *τ*; here, *τ* = 0.35:

(13) Ω_c={i|i∈Ω_bg,p_i≥τ}

With background size |*Ω*_*bg*| and selection ratio *ρ* = 0.005, the retained hard-negative count is:

(14) k=min(|Ω_c|,max(1,floor(ρ|Ω_bg|)))

For *y*_*i* = 0, *BCE*_*i* = −*log*(1 − *p*_*i*) and ∂*BCE*_*i*/∂*p*_*i* = 1/(1 − *p*_*i*) > 0, so ranking background candidates by BCE is equivalent to ranking them by *p*_*i*. The methodological distinction therefore does not lie in the ranking variable, but in restricting candidates to ground-truth background, applying a probability filter, and retaining only a small top-ranked subset.

The k candidate pixels with the largest *BCE*_*i* values are selected from *Ω*_*c* to form the hard-negative set *Ω*_*hn*:

(15) Ω_hn=TopK_{i∈Ω_c}(BCE_i,k)

HNBS Loss is the mean BCE over the selected hard-negative pixels:

(16) L_HN=(1/|Ω_hn|)Σ_{i∈Ω_hn}BCE_i

If no candidate is available, the loss returns a differentiable zero. The final objective is:

(17) L_total=0.4 L_Dice+0.3 L_Focal+0.3 L_Tversky+0.01 L_HN

The base-loss term in Equation (17) adopts L3, *L*_*base* = 0.4*L*_*Dice* + 0.3*L*_*Focal* + 0.3*L*_*Tversky*. Table 5 compares L3 with BCE (L1) and 0.5 BCE + 0.5 Dice (L2) under B1 with HNBS disabled. L3 achieved the highest Dice, IoU, and recall and was retained in the final objective. Dice loss (0.4) provides the main overlap supervision, focal loss (0.3) emphasizes difficult pixels, and Tversky loss (0.3) penalizes false negatives more strongly. HNBS Loss is added separately with λ_HNBS = 0.01.

BCE achieved the highest precision and the lowest FP area ratio, but its recall was only 0.5020 ± 0.0528. Adding Dice loss in L2 increased Dice from 0.6455 to 0.8269, IoU from 0.4870 to 0.7113, and recall from 0.5020 to 0.7766.

L3 achieved the highest Dice (0.8643 ± 0.0021), IoU (0.7717 ± 0.0047), and recall (0.8769 ± 0.0172). Relative to L2, the increases were 0.0374, 0.0604, and 0.1003, respectively, supporting the use of L3 in Equation (17).

**Figure 4:**
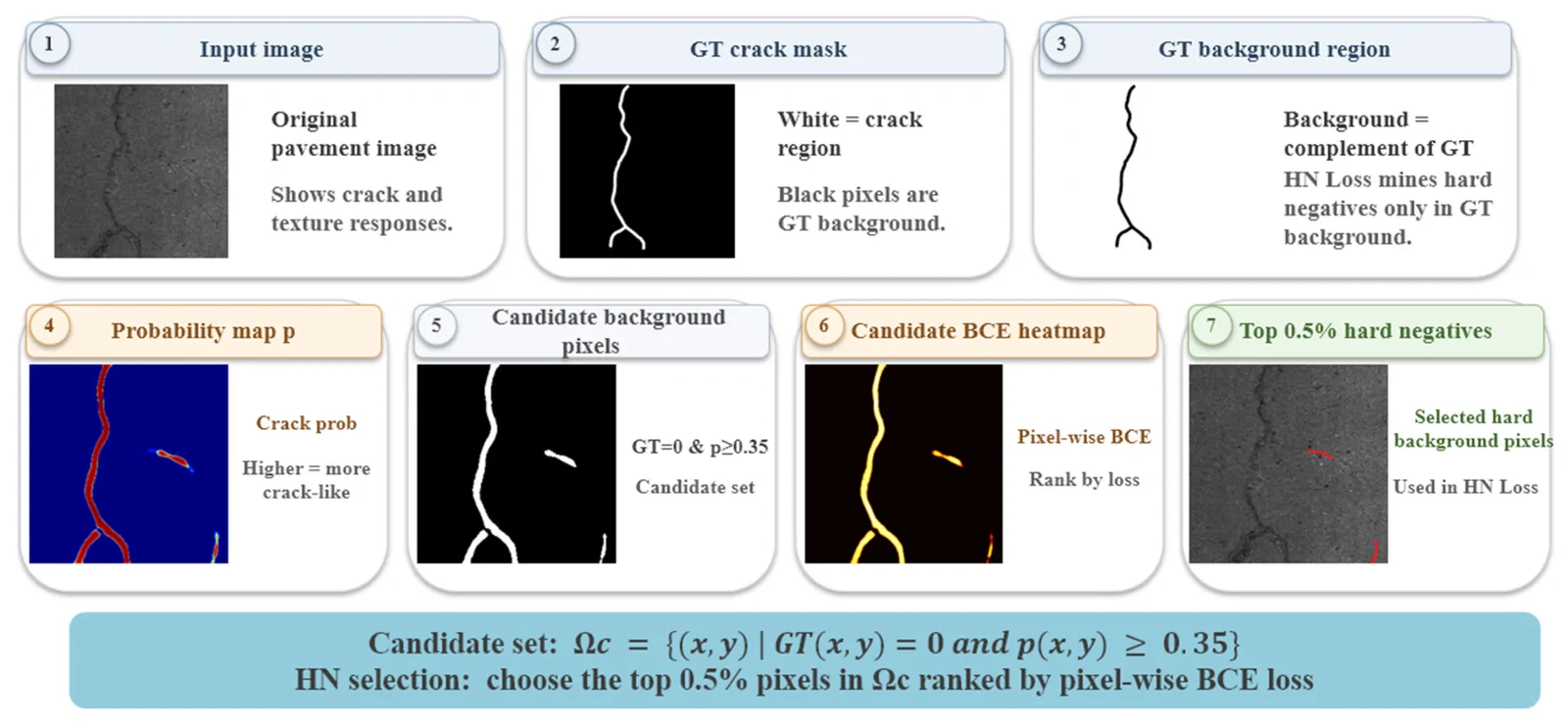
Hard-negative background selection workflow for HNBS Loss.

Figure 4 illustrates the HNBS workflow: ground-truth background pixels with *p*_*i* ≥ 0.35 are retained as candidates, ranked by BCE, and limited to the top-ranked candidates, with the maximum count set to 0.5% of the ground-truth background pixels.

#### 3.5.1. Relationship to Established Hard-Example Mining Methods

OHEM and bootstrapped cross-entropy select high-loss samples or pixels, whereas focal loss continuously reweights all pixels. HNBS uses loss-based ranking but restricts candidates to ground-truth background.

Candidates below τ are removed, and only the hardest pixels up to ratio ρ are retained. The auxiliary loss therefore focuses on confident background false positives.

Section 3.5.2 describes validation-based parameter selection, and Section 3.5.3 reports the component-wise comparison.

#### 3.5.2. Validation-Based Determination of HNBS Hyperparameters

HNBS hyperparameters were first screened using the 168 crack-containing images in the validation set because this analysis was intended specifically to compare the effect of HNBS parameter settings on crack-segmentation performance. For each configuration, the same image-level metric definitions and post-processing procedure used in the main evaluation were applied, and the resulting metrics were macro-averaged over these 168 crack-containing validation images. The checkpoint with the highest post-processed validation IoU on this subset was retained. The tested values were τ ∈ {0.25, 0.35, 0.45}, ρ ∈ {0.0025, 0.005, 0.01}, and λ_HNBS ∈ {0.005, 0.01, 0.02} (Table 6). This preliminary sensitivity analysis was conducted as a single screening run without the later fixed-seed protocol, and the test set was not used for parameter selection.

S2 (τ = 0.35, ρ = 0.005, and λ_HNBS = 0.01) achieved the best overall validation result in the preliminary screening and was therefore selected for the subsequent experiments. To assess whether this choice was sensitive to random initialization, the three leading configurations from Table 6 (S1, S2, and S4) were separately reevaluated under fixed seeds 2026, 2025, and 42 using the same 40-epoch validation and checkpoint-selection protocol. Across these three seeded runs, S1 achieved Dice = 0.5918 ± 0.0038, IoU = 0.4294 ± 0.0026, precision = 0.5166 ± 0.0228, recall = 0.7363 ± 0.0496, and FP area ratio = 0.0065 ± 0.0012; S2 achieved Dice = 0.5986 ± 0.0046, IoU = 0.4366 ± 0.0038, precision = 0.5319 ± 0.0197, recall = 0.7657 ± 0.0293, and FP area ratio = 0.0057 ± 0.0009; and S4 achieved Dice = 0.5931 ± 0.0084, IoU = 0.4309 ± 0.0084, precision = 0.5086 ± 0.0112, recall = 0.7614 ± 0.0458, and FP area ratio = 0.0071 ± 0.0009. S2 therefore retained the best mean overall validation performance across the three random initializations. These additional fixed-seed results support the robustness of the selected S2 setting across random initializations.

#### 3.5.3. Component-Wise Comparison of HNBS

Using the fixed parameters, five variants were evaluated under the B1 architecture. B1 used no auxiliary loss. Global OHEM selected the top 0.5% highest-loss pixels from the full image. Background Top-k selected the top 0.5% only from ground-truth background without a probability threshold. Background Threshold-All retained all ground-truth background pixels with *p*_*i* ≥ 0.35 without top-k truncation. Full HNBS combined ground-truth background restriction, *p*_*i* ≥ 0.35, and selection of up to the top-ranked 0.5% of ground-truth background pixels. All other settings were identical. Table 7 reports the results.

Compared with Global OHEM, Background Top-k improved Dice, IoU, precision, and recall by 0.0098, 0.0138, 0.0117, and 0.0055, respectively, and reduced the FP area ratio by 0.0005. Restricting auxiliary supervision to ground-truth background was therefore beneficial.

Background Threshold-All achieved the highest precision (0.8825 ± 0.0190) and the lowest FP area ratio (0.0039 ± 0.0009), but recall decreased to 0.8279 ± 0.0208. Full HNBS increased Dice, IoU, and recall by 0.0204, 0.0345, and 0.0455, respectively, showing that top-k selection mitigates excessive suppression.

Compared with Background Top-k, Full HNBS further improved Dice, IoU, precision, recall, and the FP area ratio. The results indicate that background restriction, probability filtering, and top-k selection make complementary contributions.

## 4. Experimental Design

### 4.1. Dataset and Annotation

**Figure 5:**
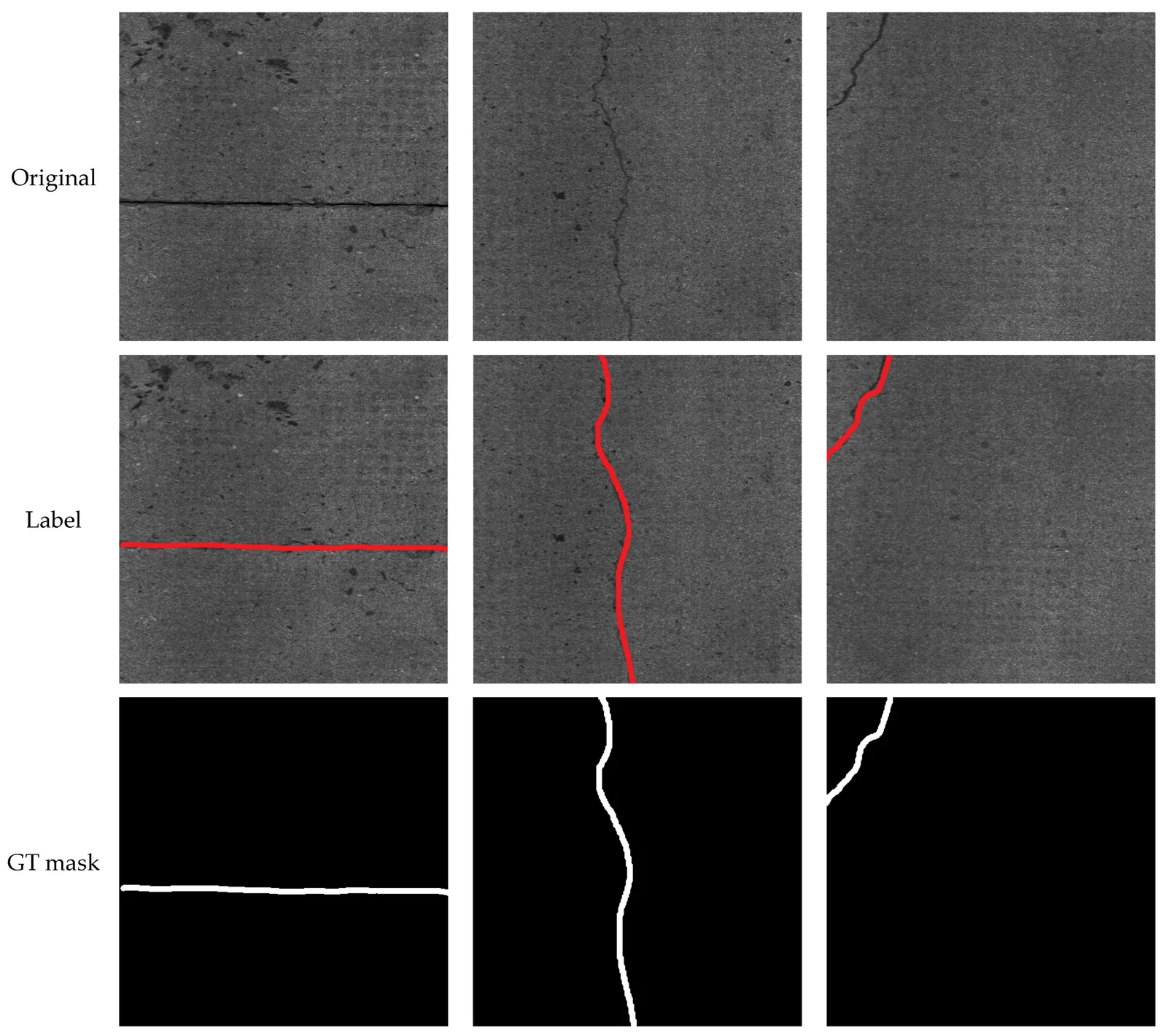
Examples of pavement images and crack annotations.

The dataset comprised 512 × 512 grayscale pavement images and annotations from the sensing system in Section 2. Crack-containing images retained realistic aggregates, voids, dark spots, tire marks, shadows, contamination, and repair textures. Red crack traces with width-adaptive thickness were converted to binary masks using Section 3.2. Images and masks were resized to 256 × 256; masks used nearest-neighbor interpolation to preserve binary labels. Examples are shown in Figure 5.

#### 4.1.1. Annotation Protocol and Quality Control

Three annotators (two postgraduates and one undergraduate) performed labeling using Adobe Photoshop 26.4. Each annotator labeled one third of the images by tracing visible crack paths and matching the brush width to the apparent crack width on the original 512 × 512 images. The other two annotators independently reviewed each image–label pair for missing segments, false markings, discontinuities, and positional errors. Disputed labels were corrected and rechecked before acceptance.

As described in Section 2.3, the 1684 training, 464 validation, and 87 test images were partitioned by acquisition day and road section, with no shared road section or continuous sequence.

The duplicate audit found no exact duplicates or confirmed cross-partition near-duplicates, so testing used mutually exclusive road sections and sessions.

The 100 crack-free images served only as a supplementary negative set and were excluded from all model-development decisions. All pixels were treated as background for false-alarm evaluation.

Figure 5 shows original grayscale images, red annotations, and binary GT masks. The slender, low-contrast cracks coexist with aggregates, voids, dark spots, and irregular texture that can resemble cracks and cause false positives, motivating CGSG and HNBS Loss.

#### 4.1.2. Public CrackForest Dataset

To provide validation on an independently acquired public road and sensor domain, the CrackForest Dataset (CFD) was additionally used [35]. CFD contains 118 pixel-level annotated RGB pavement images with a spatial resolution of 480 × 320 pixels, acquired from urban roads in Beijing using an iPhone 5. The sensor, acquisition platform, road environment, and geographic region therefore differ from the vehicle-mounted industrial-CCD field-road dataset used for the main experiments. The downloaded image–label pairs were split deterministically according to their original numerical identifiers: images 001–070 (70 images) were used for training, images 071–098 (28 images) for validation, and images 099–118 (20 images) for testing. The three partitions were mutually exclusive, with no image appearing in more than one partition. For consistency with the main experiment, CFD images were converted to grayscale, replicated across three channels, and resized to 256 × 256 pixels; binary masks were resized using nearest-neighbor interpolation.

### 4.2. Experimental Environment and Training Settings

Table 8 summarizes the experimental environment. All models used the same data split, 256 × 256 input, a batch size of 8, 40 epochs, augmentation, random seeds 42, 2025, and 2026, evaluation metrics, threshold, and post-processing. The 256 × 256 input was selected because 512 × 512 training could not be run consistently for all models with a batch size of 8 on the available 8 GB GPU and required substantially more training time. U-Net, Attention U-Net, UNet++, DeepLabV3+, SE-U-Net, and CGHN-Net were trained from scratch with Adam at 1 × 10^−4^. SegFormer-B0 and BGCrack retained their model-specific settings. The checkpoint with the highest validation IoU was retained for each model, and Table 9 summarizes the implementation details. A probability threshold of 0.5, 3 × 3 morphological opening, and removal of connected components smaller than 30 pixels were fixed on the validation set. 

**Figure 6:**
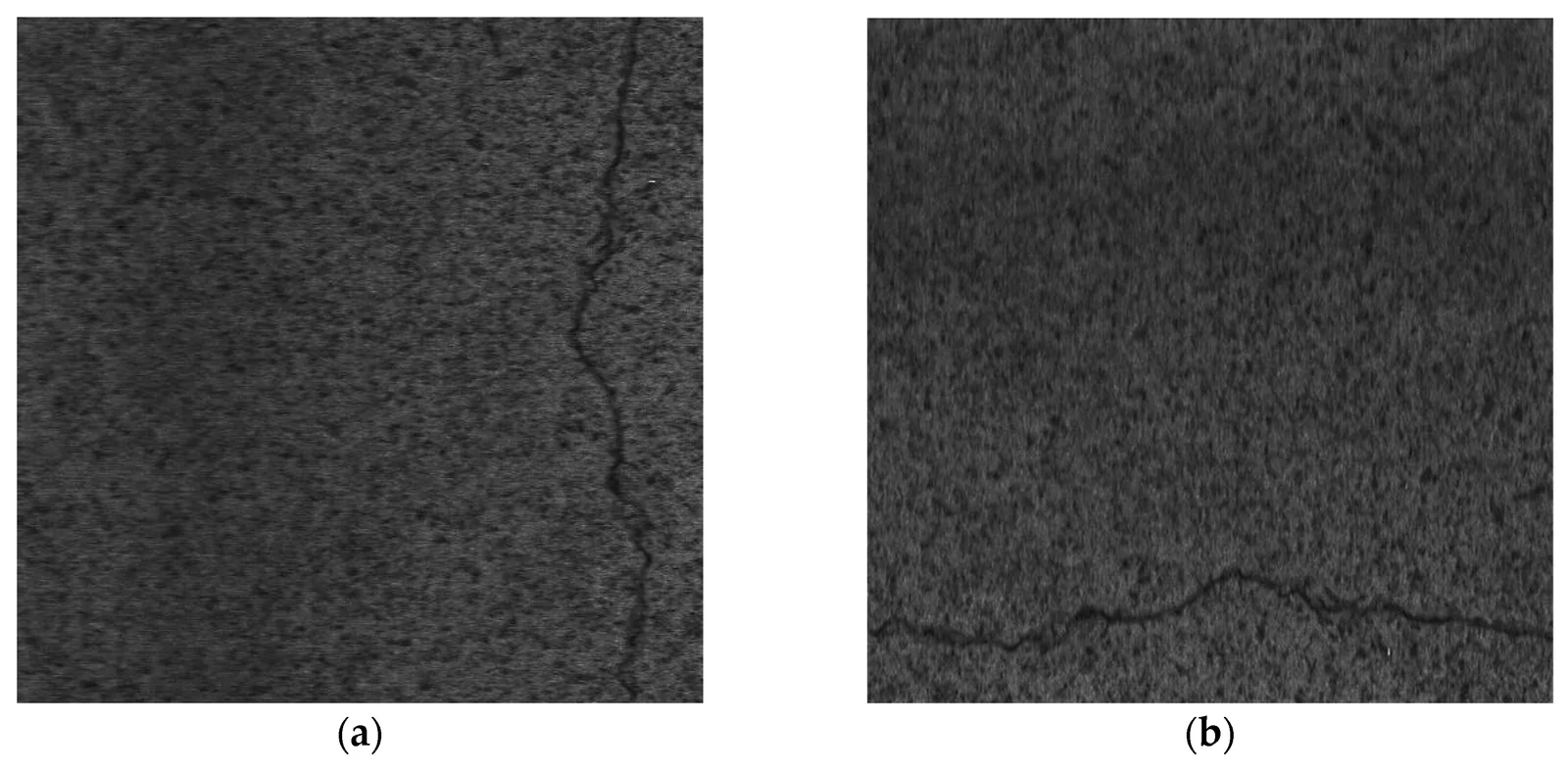

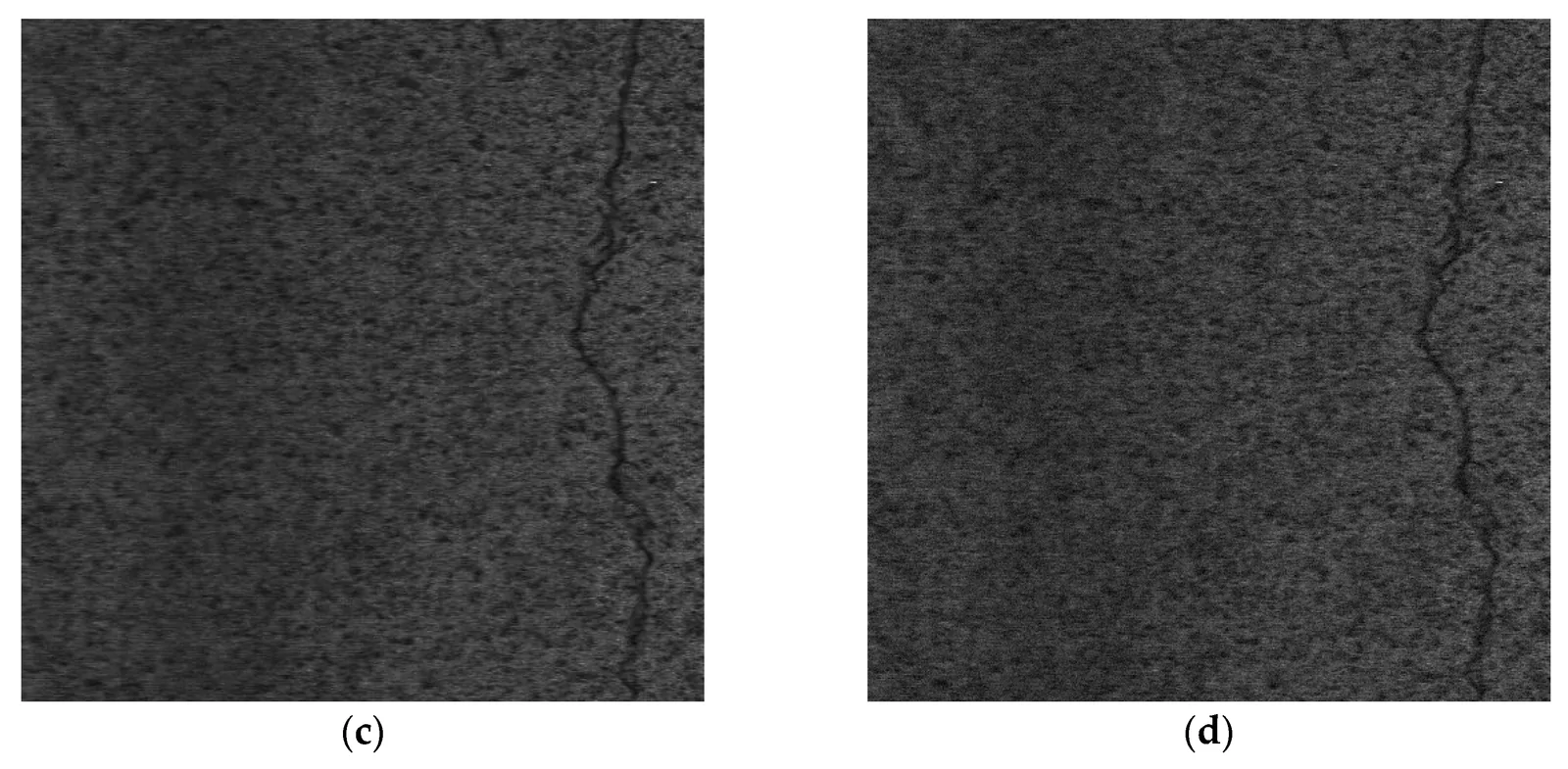
Examples of data augmentation. (**a**) Original image; (**b**) random 90° rotation; (**c**) brightness scaling (0.8–1.2); (**d**) Gaussian noise perturbation.

Figure 6 illustrates the augmentations. Rotation diversifies crack orientation, brightness scaling simulates illumination changes, and Gaussian noise improves robustness to imaging noise and pavement texture. Geometric transforms were applied synchronously to images and labels.

### 4.3. Evaluation Metrics and Statistical Analysis

Dice, IoU, precision, recall, and FP area ratio evaluated segmentation. For test image *j*, prediction $\hat{y}\text{\_}i$ and label *y*_*i* define:

(18) $$TP\;=\;\Sigma \text{\_}i\;1(\hat{y}\text{\_}i=1\;and\;y\text{\_}i=1)$$

(19) $$FP=\Sigma \text{\_}i\;1(\hat{y}\text{\_}i=1\;and\;y\text{\_}i=0)$$

(20) $$FN=\Sigma \text{\_}i\;1(\hat{y}\text{\_}i=0\;and\;y\text{\_}i=1)$$

(21) $$TN=\Sigma \text{\_}i\;1(\hat{y}\text{\_}i=0\;and\;y\text{\_}i=0)$$

*TP*, *FP*, *FN*, and *TN* denote correctly detected crack pixels, background pixels predicted as crack, missed crack pixels, and correctly classified background pixels, respectively. The metrics are as follows:

(22) Precision=TP/(TP+FP)

(23) Recall=TP/(TP+FN)

(24) Dice=2TP/(2TP+FP+FN)

(25) IoU=TP/(TP+FP+FN)

Dice and *IoU* measure overlap; precision reflects false-positive control; and recall reflects crack recovery. Higher values are better. Because false positives are central to this study, *FP* area ratio is also reported for an *H* × *W* image as follows:

(26) FP Area Ratio=FP/(H×W)

*FP area ratio* is the fraction of all pixels that are background in the ground truth but predicted as crack; lower values indicate fewer spurious regions. For routine validation and checkpoint selection, all models were evaluated on the complete validation set of 464 images, comprising 168 crack-containing images and 296 crack-free images. The metrics were first calculated independently for each image and then macro-averaged over all 464 validation images. In the implementation, ε = 1 × 10^−7^ was added to the denominators of Dice, IoU, precision, and recall. For a crack-free image, the ground truth contains no positive crack pixels, so *TP* = *FN* = 0. Under the implemented Dice and IoU definitions in Equations (24) and (25), both Dice and IoU therefore take a value of 0 for such an image, including when the predicted mask is also entirely background. Consequently, because 296 of the 464 validation images are crack-free, these zero-valued image-level scores substantially reduce the full-validation macro-averaged Dice and IoU.

This complete-validation-set protocol was used for routine validation and checkpoint selection across all models and experiments. The only exception was the HNBS parameter-sensitivity analysis in Table 6, which was intentionally evaluated on the 168 crack-containing validation images to assess the effect of HNBS parameter settings on crack-segmentation performance. The same image-level metric definitions and macro-averaging procedure were used for this subset analysis.

For the main test set, all 87 images contained annotated cracks. Dice, IoU, precision, recall, and FP area ratio were first computed separately for each test image and then macro-averaged within each random-seed run. The reported Mean ± Std values summarize these image-level macro averages across the three seeds and are not global micro-averages obtained by pooling all test pixels. Dice, IoU, precision, and recall were not used for the separate all-background crack-free subset. The *mean* and standard deviation across the three seeds are:

(27) Mean=(1/N)Σ_{s=1}^{N} x_s

(28) Std=sqrt((1/(N−1)) Σ_{s=1}^{N} (x_s−Mean)^2)

where *N* = 3 and *x*_*s* denotes the value of a given evaluation metric obtained under the *s*-th random seed.

#### Sequence-Aware Paired Statistical Procedure

The 87 test images were acquired consecutively from Road Section D, and their identifiers preserve the original acquisition order. Because neighboring images may share pavement texture, illumination, and crack morphology, they were not assumed to be mutually independent. For each image and model, each metric was first averaged across the three random-seed trials. Individual images were retained for descriptive mean differences and win rates, whereas statistical inference explicitly preserved local sequence dependence.

For Dice, IoU, precision, and recall, the paired difference was defined as CGHN-Net minus UNet++; for FP area ratio, it was defined as UNet++ minus CGHN-Net so that positive values consistently favored CGHN-Net. UNet++ was selected as the strongest conventional baseline, and all 87 image identifiers were aligned without exclusion.

Moving-block bootstrap confidence intervals were calculated from 100,000 resamples using block lengths of 3, 5, and 10 consecutive images. Block-level sign-flip tests used 1,000,000 Monte Carlo permutations for the 3-image setting and exact enumeration for the 5- and 10-image settings. Holm correction was applied across the five metrics within each block-length analysis. Per-image win rate was treated as descriptive, and statistical support required a 95% block-bootstrap confidence interval excluding zero and a Holm-adjusted *p*-value below 0.05.

For the crack-free subset, an image-level false alarm occurred when any positive pixel remained in image *j*. The false-alarm rate is as follows:

(29) $${FAR}_{image}=\frac{1}{N_{-}}\sum_{j=1}^{N_{-}}I\left(\sum_{i}{\hat{y}}_{j,i}>0\right)$$

The pixel-level false-positive area ratio on the crack-free subset is calculated as follows:

(30) $${FPAR}_{negative}=\frac{1}{N_{-}}\sum_{j=1}^{N_{-}}\frac{\sum_{i}{\hat{y}}_{j,i}}{H\times W}$$

Here, *N*_ = 100. Image-level FAR measures the frequency of false alarms, while FPAR_negative measures their mean spatial extent. Both were computed before and after the common post-processing.

### 4.4. Comparison Models and Ablation Settings

Baselines were U-Net, Attention U-Net, UNet++, DeepLabV3+, SegFormer-B0, and BGCrack. All models used the common data split, input size, training budget, random seeds, evaluation metrics, threshold, and post-processing, while retaining the architecture-specific settings listed in Table 9. Ablations were B0 (SE-U-Net), B0 + HNBS, B1 (B0 + CGSG), and CGHN-Net (B1 + HNBS). For BGCrack, epoch-wise training and validation metrics were retained for all three seeds and evaluated on the complete 464-image validation set using the same image-level macro-aggregation procedure as routine checkpoint selection. The best validation-IoU checkpoints occurred at epochs 36, 39, and 38 for seeds 42, 2025, and 2026, with validation IoUs of 0.1498, 0.1492, and 0.1495, respectively. The five highest validation-IoU values showed only small within-seed variation, with standard deviations of 0.0012, 0.0010, and 0.0006, respectively, supporting stable validation performance within the 40-epoch training budget.

For the sequence-aware paired analysis, CGHN-Net and UNet++ results were aligned by filename, averaged across seeds within each image, ordered by acquisition identifier, and analyzed using contiguous blocks of 3, 5, and 10 images without excluding any test sample.

A controlled comparison of B0, SE-U-Net with the standard attention gate, and B1 used identical backbone, loss, data, training, threshold, and post-processing settings. HNBS Loss was disabled to isolate skip gating.

Complexity was measured using a 1 × 3 × 256 × 256 input. Parameters were counted directly, GFLOPs were estimated using fvcore 0.1.5.post20221221 with FlopCountAnalysis, and FP32 inference on the RTX 4060 Ti used batch size 1, 100 warm-up iterations, and five rounds of 500 forward passes. Timing excluded data loading and post-processing. Peak GPU memory included the model, input, and activations. BGCrack was profiled with its adapted official implementation under the same hardware and profiling protocol.

HNBS parameters were selected on the validation set as described in Section 3.5.2 and then fixed at τ = 0.35, ρ = 0.005, and λ_HNBS = 0.01 for all seeded experiments.

The component variants defined in Section 3.5.3 used the same B1 architecture, L3 base loss, data split, training schedule, random seeds, checkpoint criterion, threshold, and post-processing.

The crack-free evaluation used validation-selected checkpoints of B0, UNet++, B1, B1+OHEM-BCE, and CGHN-Net for seeds 2026, 2025, and 42. The same threshold and post-processing were applied without retraining or adjustment. Image-level FAR and negative-set FP area ratio were reported as Mean ± Std.

The base-loss comparison used B1 with HNBS disabled and compared BCE (L1), 0.5 BCE + 0.5 Dice (L2), and 0.4 Dice + 0.3 Focal + 0.3 Tversky (L3) under identical settings.

For the public-dataset experiment, UNet++, BGCrack, and CGHN-Net were retrained using the fixed CFD split defined in Section 4.1.2: images 001–070 for training (*n* = 70), 071–098 for validation (*n* = 28), and 099–118 for testing (*n* = 20). The three models were run under seeds 2026, 2025, and 42. The common input, threshold, post-processing, checkpoint criterion, and metrics were retained, together with the model-specific optimization settings in Table 9.

## 5. Experimental Results and Analysis

### 5.1. Comparison with Representative Semantic Segmentation Models

Table 10 reports Mean ± Std across the three random seeds. CGHN-Net achieved the highest Dice (0.8682 ± 0.0055), IoU (0.7791 ± 0.0102), and recall (0.8734 ± 0.0266), with precision of 0.8780 ± 0.0161 and FP area ratio of 0.0042 ± 0.0008.

Relative to U-Net, CGHN-Net improved Dice, IoU, precision, and recall and reduced the FP area ratio from 0.0053 to 0.0042.

Compared with UNet++, CGHN-Net improved Dice, IoU, precision, and recall by 0.0092, 0.0142, 0.0098, and 0.0084, respectively, while reducing the FP area ratio from 0.0047 to 0.0042.

SegFormer-B0 achieved the highest precision (0.9489 ± 0.0064) and the lowest FP area ratio (0.0008 ± 0.0002) among the field-road comparison models, but its recall was only 0.4523 ± 0.0355, resulting in substantially lower Dice and IoU.

BGCrack achieved higher precision (0.8853 ± 0.0071) and a lower FP area ratio (0.0033 ± 0.0004) than CGHN-Net, but lower Dice, IoU, and recall, indicating a more conservative prediction pattern.

Table 11 compares all seven models under the matched profiling protocol. BGCrack had the fewest parameters, SegFormer-B0 had the lowest reported GFLOPs, DeepLabV3+ was fastest, and U-Net used the least peak GPU memory. CGHN-Net achieved 146.37 FPS.

Compared with UNet++, CGHN-Net used 11.4% fewer parameters, 46.1% fewer GFLOPs, and 44.2% lower latency, increasing speed from 81.69 to 146.37 FPS. Its complexity was close to that of Attention U-Net while providing better field-road segmentation performance.

### 5.2. Additional Validation on the Public CFD

Table 12 reports the three-seed results after retraining UNet++, BGCrack, and CGHN-Net using the fixed 70/28/20 CFD training, validation, and test split described in Section 4.1.2.

CGHN-Net achieved the highest CFD Dice (0.6679 ± 0.0162), IoU (0.5028 ± 0.0182), and recall (0.9486 ± 0.0085). Relative to UNet++, Dice and IoU increased by 0.0296 and 0.0268, and recall increased by 0.0505.

BGCrack achieved the highest CFD precision and the lowest FP area ratio. Thus, CGHN-Net provided the strongest overlap and crack recovery, but not the best result for every error-control metric.

### 5.3. Sequence-Aware Paired Statistical Analysis

Table 13 reports the sequence-aware paired comparison with UNet++ using moving-block bootstrap confidence intervals and block-level sign-flip tests for block lengths of 3, 5, and 10 images.

Positive values favor CGHN-Net; FP area ratio reduction was defined as UNet++ minus CGHN-Net. Mean paired differences and per-image win rates are descriptive and are shown once for each metric. Confidence intervals used 100,000 moving-block bootstrap resamples. Sign-flip tests used 1,000,000 Monte Carlo permutations for 3-image blocks and exact enumeration for 5- and 10-image blocks.

All five mean differences remained positive, and every 95% block-bootstrap confidence interval excluded zero under block lengths of 3, 5, and 10 images. Dice and IoU gains were 0.0102 and 0.0154, with per-image win rates of 67.8% and 69.0%. Under the conservative 10-image setting, their Holm-adjusted *p*-values were both 0.0195.

Precision and recall increased by 0.0105 and 0.0092, with win rates of 58.6% and 64.4%. Their Holm-adjusted *p*-values remained below 0.05 for all block lengths and reached 0.0469 under the 10-image setting, indicating statistical support that is more cautious than the original independent-image analysis.

FP area ratio decreased by 0.00055, with a 56.3% per-image win rate. The block-bootstrap intervals remained above zero, and the Holm-adjusted *p*-value was 0.0469 for 10-image blocks. These results support within-section consistency after accounting for plausible local spatial and sequential dependence; they do not establish independence or generalization across road sections, sensors, or acquisition domains.

### 5.4. Effect of the Common Post-Processing Procedure

Raw thresholded outputs of CGHN-Net and UNet++ were evaluated before morphology and component filtering (Table 14).

Before post-processing, CGHN-Net exceeded UNet++ in Dice, IoU, precision, and recall and reduced the FP area ratio by 14.3%.

For CGHN-Net, the complete post-processing pipeline increased Dice and IoU by 0.0032 and 0.0059, changed precision and recall by −0.0010 and −0.0018, and left the FP area ratio unchanged to four decimal places. Raw CGHN-Net also outperformed post-processed UNet++ on every metric, indicating that post-processing had only a limited influence on the reported advantage.

### 5.5. False-Positive Evaluation on the Held-Out Crack-Free Subset

The 100-image crack-free subset tested whether difficult textures alone triggered crack predictions. Since every pixel was background, any positive prediction was a false alarm. Table 15 reports image-level FAR and FP area ratio before and after post-processing.

CGHN-Net achieved the lowest values for all four measures. Raw image-level FAR and FP area ratio were 0.1866 ± 0.0723 and 0.0019 ± 0.0006; post-processing reduced them to 0.1300 ± 0.0335 and 0.0018 ± 0.0006.

Relative to UNet++, CGHN-Net reduced raw and post-processed FAR by 22.3% and 26.4%, and reduced raw and post-processed FP area ratios by 17.4% and 14.3%. Relative to B1, post-FAR and FP area ratios fell by 58.1% and 51.4%.

OHEM-BCE did not improve B1 on the negative set, whereas CGHN-Net reduced post FAR and FP area ratios by 54.1% and 53.8% relative to OHEM-BCE. Background-restricted mining was therefore better aligned with texture-induced false alarms.

**Figure 7:**
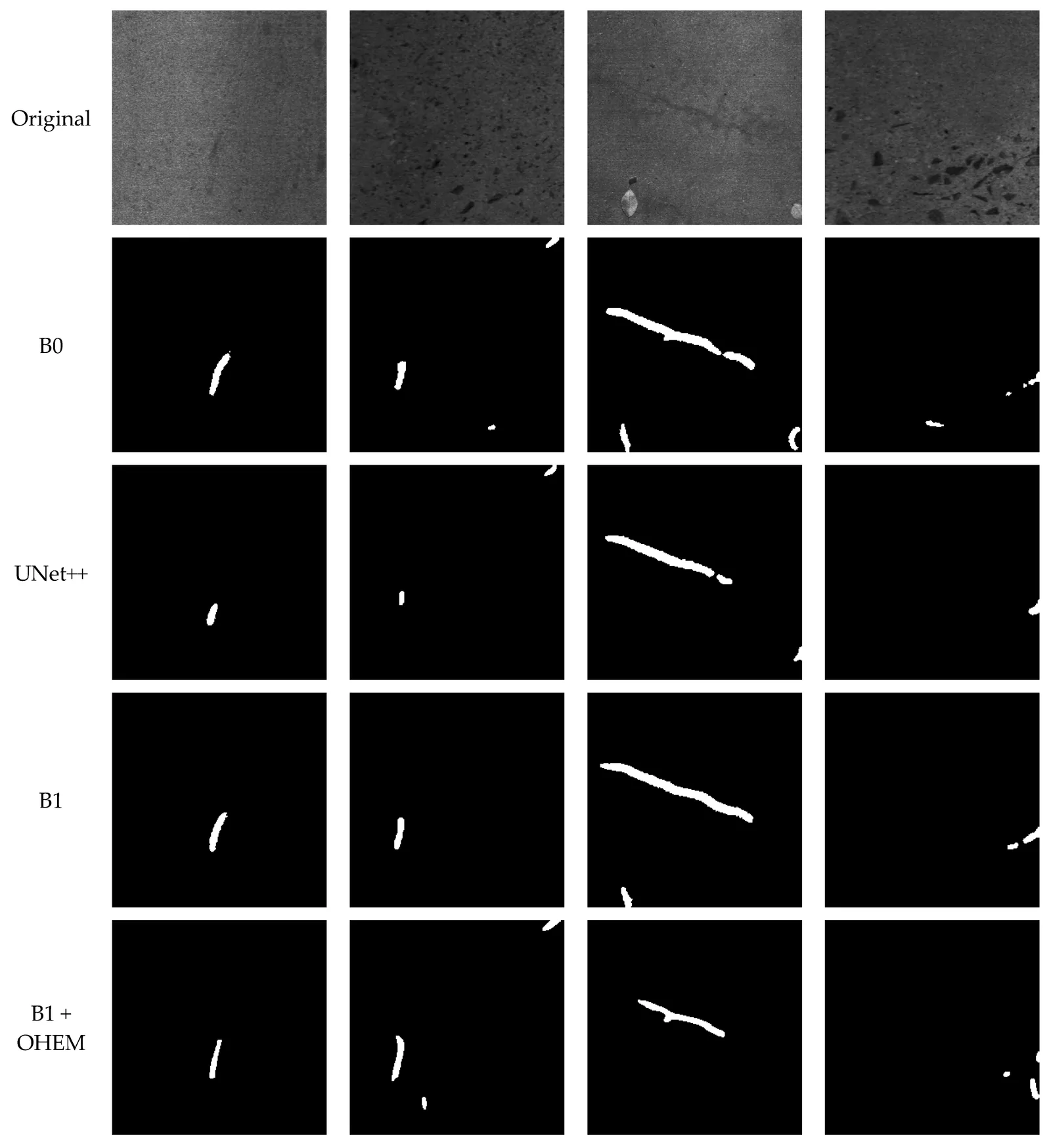

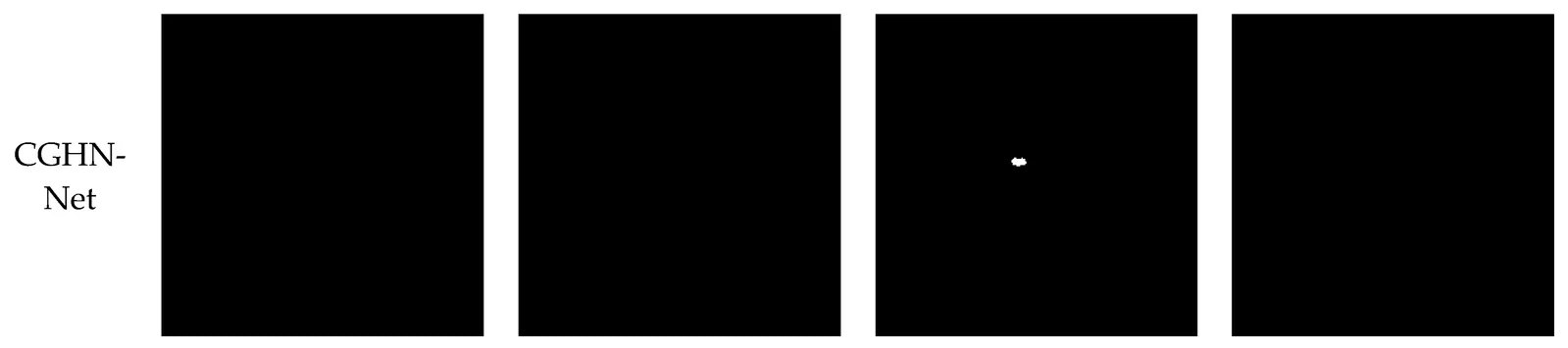
Representative false-positive predictions on crack-free images from the held-out Road Section D subset.

Figure 7 compares four crack-free images. All white regions are false alarms. B0, UNet++, B1, and OHEM-BCE produce isolated or elongated responses, whereas CGHN-Net removes nearly all of them, consistent with Table 15.

### 5.6. Ablation Experiment

All four ablations were trained with seeds 2026, 2025, and 42 under identical settings. Table 16 reports the Mean ± Std across the three runs. B0 is SE-U-Net, B0+HNBS adds only HNBS Loss, B1 adds CGSG, and CGHN-Net combines B1 with HNBS Loss.

B0 achieved Dice 0.8624 ± 0.0063, IoU 0.7708 ± 0.0105, precision 0.8624 ± 0.0189, recall 0.8796 ± 0.0280, and FP area ratio 0.0049 ± 0.0007.

CGSG alone increased Dice, IoU, and precision and reduced the FP area ratio to 0.0047 ± 0.0005, with only a small decrease in recall.

HNBS alone increased precision and reduced the FP area ratio but decreased Dice, IoU, and recall.

CGHN-Net achieved the best mean Dice, IoU, and precision and the lowest FP area ratio among the four ablations, while retaining recall of 0.8734 ± 0.0266.

The ablation results support the combined use of CGSG for feature filtering and HNBS for suppressing the remaining elevated-probability background errors.

### 5.7. Qualitative Result Analysis

**Figure 8:**
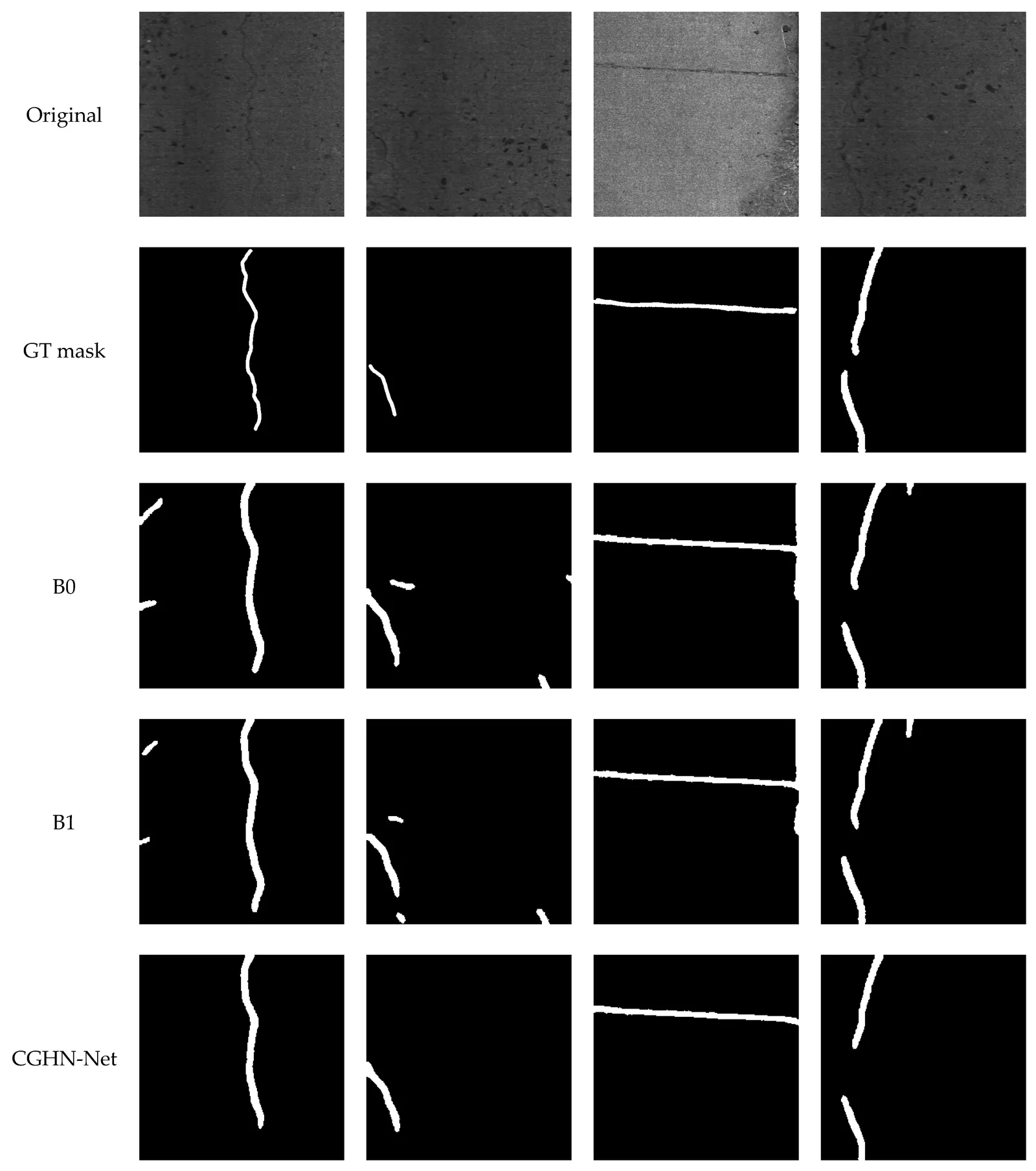
Comparison of crack prediction results obtained by different models.

**Figure 9:**
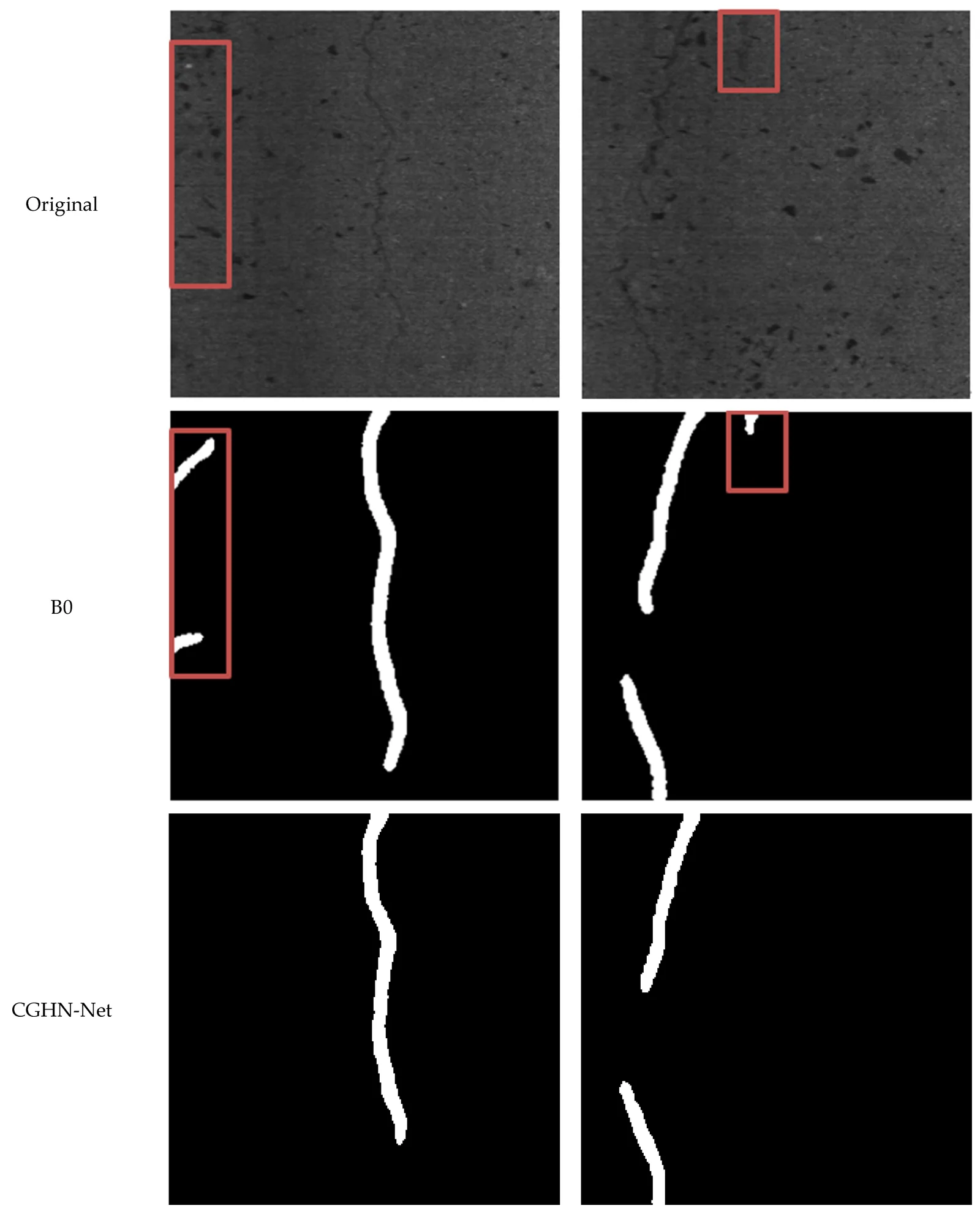
Local comparison of texture-induced false-positive suppression. Red boxes indicate representative texture-induced false-positive regions.

Figure 8 and Figure 9 compare B0, B1, and CGHN-Net on representative texture-induced false positives.

Figure 8 shows that CGSG reduces false responses and that CGHN-Net further suppresses elevated-probability background predictions while preserving the main cracks, consistent with Table 10.

Figure 9 enlarges dark particles, local edges, and weak textures that resemble cracks. CGHN-Net suppresses the false responses produced by B0 while preserving the principal crack regions.

### 5.8. Discussion

Field-road crack segmentation remains challenging because slender and low-contrast cracks often share similar local grayscale, edge, and linear characteristics with coarse aggregates, dark spots, void boundaries, tire marks, shadows, and repair textures. These background structures may be transferred through encoder skip connections and reconstructed by the decoder as false crack responses. Therefore, effective crack segmentation in this setting requires not only the preservation of weak spatial details, but also the suppression of texture features that are visually similar to cracks.

The controlled comparison with the standard attention gate clarifies the role of CGSG. Both methods use decoder semantics to reweight encoder skip features, but the standard gate performs attention estimation after spatial downsampling, whereas CGSG operates directly at the native resolution of each skip connection using the already upsampled decoder feature. The standard gate achieved stronger background suppression, as reflected by its higher precision and lower FP area ratio, but its recall decreased substantially, indicating that some weak crack responses were also removed. In contrast, CGSG retained a recall of 0.8769 ± 0.0172 and achieved higher Dice and IoU than the standard gate, while using fewer parameters and slightly lower measured latency. These results indicate that the main advantage of CGSG lies in achieving a better balance between texture suppression and weak-crack preservation rather than simply maximizing background suppression.

The HNBS component comparison further explains why conventional global hard-example mining was not sufficient for this task. Global OHEM selected the highest-loss pixels from all valid pixels and therefore allowed crack pixels, uncertain boundary pixels, and background pixels to compete within the same candidate pool. Restricting the candidate set to ground-truth background pixels improved Dice, IoU, precision, and recall, showing that the auxiliary loss became better aligned with the intended objective of suppressing texture-induced false positives.

The two additional intermediate variants clarify the roles of probability filtering and top-k selection. Background Top-k used ground-truth background restriction and top-0.5% selection but did not apply the probability threshold. Full HNBS further restricted these candidates to pixels with *p*_*i* ≥ 0.35, improving all five reported metrics relative to Background Top-k. This result indicates that probability filtering excludes low-probability background pixels from the auxiliary-loss candidate set and concentrates supervision on elevated-probability background responses.

Background Threshold-All used ground-truth background restriction and *p*_*i* ≥ 0.35, but applied the loss to all eligible candidates without top-k truncation. It achieved the highest precision and the lowest FP area ratio, but its recall decreased to 0.8279 ± 0.0208. After retaining up to the top-ranked 0.5% of ground-truth background pixels among the threshold-qualified candidates, full HNBS increased Dice, IoU, and recall by 0.0204, 0.0345, and 0.0455, respectively. Thus, top-k selection limits the auxiliary loss to a small subset of background regions and reduces the tendency toward overly conservative crack predictions. Taken together, background restriction defines the appropriate candidate domain, probability filtering restricts the candidate set to background pixels with elevated predicted crack probabilities, and top-k selection limits the auxiliary supervision to the most severe errors. The component results therefore support complementary contributions from the three design choices.

The ablation experiment also shows that HNBS is most effective when combined with CGSG. HNBS alone improved precision and reduced the FP area ratio relative to the SE-U-Net backbone, but it also decreased Dice, IoU, and recall. This indicates that loss-level suppression alone cannot fully resolve feature confusion when texture-dominated encoder features are still directly transferred to the decoder. CGSG first reduces the transmission of irrelevant texture information, after which HNBS focuses optimization on the remaining elevated-probability background errors. Their combination produced the best overall ablation result, supporting the intended feature-level and optimization-level cooperation.

Across three random seeds, CGHN-Net achieved the highest mean Dice, IoU, and recall among all evaluated field-road models. SegFormer-B0 and BGCrack obtained lower FP area ratios than CGHN-Net, but both showed lower recall. SegFormer-B0 was the most conservative baseline, achieving the highest precision and lowest FP area ratio but a recall of only 0.4523 ± 0.0355, which resulted in much lower Dice and IoU. BGCrack retained higher recall than SegFormer-B0 but still remained below CGHN-Net in Dice, IoU, and recall. CGHN-Net maintained a more balanced precision–recall relationship and therefore provided stronger overall crack-region overlap rather than optimizing only one error-control metric.

The sequence-aware paired analysis strengthens this conclusion by considering the local dependence between neighboring test images. Positive paired differences over UNet++ were retained for Dice, IoU, precision, recall, and FP area ratio reduction under block lengths of 3, 5, and 10 consecutive images. All moving-block bootstrap confidence intervals excluded zero, and the Holm-adjusted *p*-values remained below 0.05. These results indicate that the observed improvements were not limited to a few isolated test images and remained statistically supported after accounting for plausible spatial and sequential correlation within the acquisition sequence.

The crack-free subset provided a direct evaluation of texture-induced false alarms without interference from true crack regions. CGHN-Net achieved the lowest raw and post-processed image-level false-alarm rates and FP area ratios among the evaluated models. The raw-output comparison also showed that CGHN-Net outperformed UNet++ before morphological opening and connected-component removal. Therefore, the observed improvement mainly originates from the learned feature filtering and hard-negative supervision rather than from post-processing alone.

The CFD experiment provides additional evidence that the proposed strategy remains effective on a public dataset acquired using a different sensor, platform, road environment, and geographic region. After retraining each model on mutually exclusive CFD training and validation partitions, CGHN-Net achieved the highest Dice, IoU, and recall among UNet++, BGCrack, and CGHN-Net. BGCrack retained the highest precision and lowest FP area ratio, indicating stronger conservative suppression, whereas CGHN-Net provided better crack recovery and overall overlap. Because the models were retrained on CFD, this experiment provides independent public-dataset validation after retraining rather than zero-shot cross-domain transfer.

Changes in sensor response, pavement material, illumination, and acquisition conditions can alter grayscale and background texture distributions. CGSG does not rely on fixed grayscale or edge thresholds; after retraining, its decoder-guided reweighting can remain applicable when crack and background appearances change. HNBS similarly defines candidate hard negatives from ground-truth background pixels and predicted crack probabilities rather than predefined texture classes, allowing it to continue targeting elevated-probability background responses in a new training domain. Related remote-sensing detection research has also shown that contextual feature enhancement and attention can reduce complex-background interference [36]. The present CFD results therefore support the applicability of the complete CGHN-Net after retraining, but do not establish zero-shot invariance of CGSG or HNBS.

The 256 × 256 input resolution was selected as a practical compromise. Training all comparison models at 512 × 512 with a batch size of 8 could not be performed consistently on the available 8 GB GPU and would have substantially increased the time required for the complete three-seed protocol. The lower resolution enabled a matched training budget across all models, although extremely thin cracks may lose some apparent width or boundary detail. Similarly, the 30-pixel connected-component threshold was only used to remove small isolated responses. The post-processing comparison shows that the complete pipeline only produced small changes in Dice, IoU, precision, recall, and FP area ratio. Therefore, this threshold did not determine the reported model ranking or the principal performance gains.

Several limitations remain. The CFD experiment involved retraining and therefore does not establish zero-shot transfer from the field-road dataset. Only one external public dataset was evaluated, and broader validation across additional road materials, illumination conditions, acquisition platforms, and sensors is still required. The 256 × 256 input may reduce the representation of extremely thin cracks, and higher-resolution training should be examined when larger-memory hardware is available. Finally, the present study addresses binary crack segmentation and does not distinguish crack types or other pavement distresses.

## 6. Conclusions

(1) CGSG applies additive semantic gating directly at the native resolution of all three encoder skip connections. Compared with the standard attention gate under identical training conditions, CGSG improved Dice, IoU, and recall by 0.0240, 0.0360, and 0.0557, respectively. The standard gate provided stronger background suppression but removed more weak crack responses, whereas CGSG achieved a better balance between texture filtering and crack-detail preservation with comparable computational efficiency.
(2) HNBS restricts hard-pixel mining to ground-truth background pixels, applies a probability threshold of *p*_*i* ≥ 0.35, and retains up to the top-ranked 0.5% of ground-truth background pixels for auxiliary BCE supervision. Component-wise comparison showed that background restriction improved the result relative to global OHEM, probability filtering further focused supervision on elevated-probability background responses, and top-k selection mitigated excessive suppression when many threshold-qualified background pixels were present. These three components made complementary contributions to the complete HNBS formulation.
(3) Combining CGSG and HNBS produced the best overall ablation result. Under three random seeds, CGHN-Net achieved Dice, IoU, precision, recall, and FP area ratio values of 0.8682 ± 0.0055, 0.7791 ± 0.0102, 0.8780 ± 0.0161, 0.8734 ± 0.0266, and 0.0042 ± 0.0008, respectively. It achieved the highest mean Dice, IoU, and recall among the evaluated field-road models, while sequence-aware comparison with UNet++ remained statistically supported under block lengths of 3, 5, and 10 images. On the 100-image crack-free subset, CGHN-Net also achieved the lowest post-processed false-alarm rate and FP area ratio among the included models.
(4) After three-seed retraining on the public CFD, CGHN-Net achieved Dice, IoU, and recall values of 0.6679 ± 0.0162, 0.5028 ± 0.0182, and 0.9486 ± 0.0085, respectively, which were the highest among UNet++, BGCrack, and CGHN-Net. BGCrack achieved the highest precision and lowest FP area ratio, whereas CGHN-Net provided stronger crack recovery and overall overlap. This experiment provides additional validation on an independently acquired public road and sensor dataset after retraining, but broader zero-shot and multi-domain evaluation remains necessary.

## Figures and Tables

**Table 1:** Main specifications of the vehicle-mounted sensing and image-acquisition system.

Item	Configuration
Acquisition platform	Vehicle-mounted multifunctional pavement inspection system
Vehicle platform	Modified Ford Transit
Imaging sensor	Industrial-grade CCD camera
Additional sensors	Three-dimensional laser scanner, GPS, and IMU
Pavement measurement resolution	Approximately 1 mm
Image type used in this study	Two-dimensional grayscale pavement image
Original image size used in the dataset	512 × 512 pixels
Network input size	256 × 256 pixels
Operating speed	Approximately 5–30 km/h
Speed in crack-dense areas	Approximately 5–15 km/h
Sampling interval in crack-dense areas	Approximately 10 cm
Sampling interval on regular sections	Approximately 20 cm
Glare suppression	Polarizing filter
Exposure control	Dynamic exposure adjustment
Frame quality control	Frames with more than 30% occlusion were excluded or reacquired
Acquisition region	High-standard farmland field roads in Huzhou, China
Acquisition sessions	Three independent days
Dataset partition	Different acquisition days and mutually exclusive road sections

**Table 2:** Operation-by-operation comparison between the standard attention gate and CGSG.

Comparison Item	Standard Attention Gate	CGSG
Encoder input	High-resolution skip feature	High-resolution skip feature
Decoder input	Lower-resolution decoder feature	Already upsampled decoder feature
Encoder projection	2 × 2, stride-2 Conv + BN	1 × 1 Conv + BN
Decoder projection	1 × 1 Conv + BN	1 × 1 Conv + BN
Feature fusion	Element-wise addition	Element-wise addition
Nonlinearity	ReLU	ReLU
Attention generation	1 × 1 Conv + Sigmoid	1 × 1 Conv + Sigmoid
Internal skip downsampling	Required	Not required
Attention-map upsampling	Required	Not required
Output	Original skip weighted by attention map	Original skip weighted by attention map
Deployment	Three controlled skip gates	Three same-resolution skip gates
Primary objective	Generic spatial selection	Texture suppression with weak-crack preservation

**Table 3:** Controlled segmentation comparison of B0, the standard attention gate, and CGSG.

Model	Dice	IoU	Precision	Recall	FP Area Ratio
B0: SE-U-Net	0.8624 ± 0.0063	0.7708 ± 0.0105	0.8624 ± 0.0189	0.8796 ± 0.0280	0.0049 ± 0.0007
SE-U-Net + Standard AG	0.8403 ± 0.0058	0.7357 ± 0.0085	0.8785 ± 0.0164	0.8212 ± 0.0149	0.0040 ± 0.0008
B1: SE-U-Net + CGSG	0.8643 ± 0.0021	0.7717 ± 0.0047	0.8670 ± 0.0125	0.8769 ± 0.0172	0.0047 ± 0.0005

**Table 4:** Model complexity and inference speed of the controlled variants.

Model	Params (M)	GFLOPs	Time (ms/Image)	FPS
B0: SE-U-Net	7.7114	36.8270	12.6682 ± 0.6023	78.94
SE-U-Net + Standard AG	7.9706	37.4360	13.9419 ± 0.2864	71.73
B1: SE-U-Net + CGSG	7.8121	37.6507	13.7077 ± 0.2623	72.95

**Table 5:** Controlled comparison of base loss functions under the B1 architecture (mean ± standard deviation).

Base Loss	Dice	IoU	Precision	Recall	FP Area Ratio
L1: BCE	0.6455 ± 0.0491	0.4870 ± 0.0500	0.9554 ± 0.0028	0.5020 ± 0.0528	0.0008 ± 0.0002
L2: 0.5 BCE + 0.5 Dice	0.8269 ± 0.0124	0.7113 ± 0.0200	0.9014 ± 0.0206	0.7766 ± 0.0363	0.0030 ± 0.0008
L3: 0.4 Dice + 0.3 Focal + 0.3 Tversky	0.8643 ± 0.0021	0.7717 ± 0.0047	0.8670 ± 0.0125	0.8769 ± 0.0172	0.0047 ± 0.0005

**Table 6:** Forty-epoch results on the crack-containing validation subset (*n* = 168) for the preliminary HNBS hyperparameter sensitivity analysis.

Config	τ	ρ	λ_HNBS	Dice	IoU	Precision	Recall	FP Area Ratio
S2	0.35	0.0050	0.010	0.6025	0.4397	0.5547	0.7971	0.0047
S1	0.25	0.0050	0.010	0.5942	0.4285	0.5429	0.6800	0.0051
S4	0.35	0.0025	0.010	0.5834	0.4212	0.5211	0.7088	0.0061
S7	0.35	0.0050	0.020	0.5813	0.4160	0.4737	0.7876	0.0076
S3	0.45	0.0050	0.010	0.5750	0.4131	0.4777	0.7673	0.0077
S6	0.35	0.0050	0.005	0.5656	0.4105	0.5055	0.6970	0.0060
S5	0.35	0.0100	0.010	0.5625	0.4098	0.5115	0.6917	0.0055

**Table 7:** HNBS component comparison under B1 (mean ± standard deviation).

Variant	Dice	IoU	Precision	Recall	FP Area Ratio
B1 (no auxiliary loss)	0.8643 ± 0.0021	0.7717 ± 0.0047	0.8670 ± 0.0125	0.8769 ± 0.0172	0.0047 ± 0.0005
Global OHEM	0.8453 ± 0.0067	0.7442 ± 0.0114	0.8522 ± 0.0137	0.8553 ± 0.0210	0.0053 ± 0.0008
Background Top-k	0.8551 ± 0.0037	0.7580 ± 0.0075	0.8639 ± 0.0132	0.8608 ± 0.0080	0.0048 ± 0.0008
Background Threshold-All	0.8478 ± 0.0077	0.7446 ± 0.0124	0.8825 ± 0.0190	0.8279 ± 0.0208	0.0039 ± 0.0009
Full HNBS	0.8682 ± 0.0055	0.7791 ± 0.0102	0.8780 ± 0.0161	0.8734 ± 0.0266	0.0042 ± 0.0008

**Table 8:** Experimental environment.

Item	Configuration
Operating System	Windows-11-10.0.22631-SP0
Python	3.12.0
PyTorch/Torchvision	2.2.0+cu118/0.17.0+cu118
CUDA/cuDNN	CUDA 11.8/cuDNN 8700
GPU	NVIDIA GeForce RTX 4060 Ti, 8.0 GB
CPU	Intel64 Family 6 Model 151 Stepping 2, GenuineIntel
OpenCV/NumPy/Pandas/Pillow	OpenCV 4.13.0; NumPy 1.26.4; Pandas 2.2.3; Pillow 11.1.0

**Table 9:** Concise implementation details of the comparison and ablation models.

Model	Encoder and Initialization	Optimization and Objective	Regularization and Checkpoint
U-Net	Encoder channels 64/128/256 with a 512-channel bottleneck; random initialization	Adam, 1 × 10^−4^, fixed learning rate; Dice–Focal–Tversky loss	No weight decay or dropout; highest validation IoU
Attention U-Net	Same encoder as U-Net with additive gates at three skip connections; random initialization	Adam, 1 × 10^−4^, fixed learning rate; Dice–Focal–Tversky loss	No weight decay or dropout; highest validation IoU
UNet++	Feature channels 64/128/256/512 with nested dense skip connections; random initialization	Adam, 1 × 10^−4^, fixed learning rate; Dice–Focal–Tversky loss	No weight decay or dropout; highest validation IoU
DeepLabV3+	Residual encoder channels 64/128/192/256 and ASPP rates 6/12/18; random initialization	Adam, 1 × 10^−4^, fixed learning rate; Dice–Focal–Tversky loss	ASPP dropout 0.1, weight decay 0; highest validation IoU
SegFormer-B0	MiT-B0 depths 2/2/2/2 and dimensions 32/64/160/256; ImageNet-1K encoder and random two-class head	Cross-entropy; AdamW, 6 × 10^−5^ encoder and 6 × 10^−4^ head; 1500-iteration warm-up and Poly decay	Weight decay 0.01 excluding normalization parameters and biases; highest validation IoU
BGCrack	Official BGCrack network; random initialization; input normalized from [0, 1] to [−1, 1]; resolution-dependent HFIE modules adapted from 128 × 128/64 × 64 to 64 × 64/32 × 32 for the 256 × 256 input; remaining architecture unchanged.	Adam, 6 × 10^−3^, fixed learning rate; official segmentation, boundary, and gradient joint loss	Weight decay 0; highest validation IoU
SE-U-Net	SE encoder channels 64/128/256 with a 512-channel bottleneck; CGHN-Net adds CGSG; random initialization	Adam, 1 × 10^−4^, fixed learning rate; Dice–Focal–Tversky loss, plus HNBS for CGHN-Net	No weight decay or dropout; highest validation IoU

**Table 10:** Test-set crack segmentation performance of different models (mean ± standard deviation).

Model	Dice	IoU	Precision	Recall	FP Area Ratio
U-Net	0.8568 ± 0.0047	0.7605 ± 0.0064	0.8580 ± 0.0098	0.8723 ± 0.0162	0.0053 ± 0.0005
Attention U-Net	0.8566 ± 0.0084	0.7611 ± 0.0143	0.8630 ± 0.0109	0.8663 ± 0.0216	0.0050 ± 0.0005
UNet++	0.8590 ± 0.0107	0.7649 ± 0.0170	0.8682 ± 0.0041	0.8650 ± 0.0258	0.0047 ± 0.0002
DeepLabV3+	0.8355 ± 0.0069	0.7268 ± 0.0107	0.8508 ± 0.0071	0.8395 ± 0.0125	0.0053 ± 0.0003
SegFormer-B0	0.5895 ± 0.0342	0.4381 ± 0.0323	0.9489 ± 0.0064	0.4523 ± 0.0355	0.0008 ± 0.0002
BGCrack	0.7989 ± 0.0052	0.6738 ± 0.0067	0.8853 ± 0.0071	0.7409 ± 0.0065	0.0033 ± 0.0004
CGHN-Net	0.8682 ± 0.0055	0.7791 ± 0.0102	0.8780 ± 0.0161	0.8734 ± 0.0266	0.0042 ± 0.0008

**Table 11:** Model complexity, peak GPU memory, and inference speed under the matched profiling protocol and hardware environment.

Model	Params (M)	GFLOPs	Peak GPU Memory (MB)	Time (ms/Image)	FPS
U-Net	7.7002	36.8197	152.94	5.6853 ± 0.1451	175.89
Attention U-Net	7.7873	37.6434	237.25	6.4305 ± 0.0983	155.51
UNet++	8.8196	69.8792	261.50	12.2416 ± 0.0013	81.69
DeepLabV3+	5.9269	10.1004	158.67	1.8506 ± 0.0315	540.38
SegFormer-B0	3.7147	1.7739	189.80	5.2831 ± 0.9826	189.28
BGCrack	2.3153	3.3814	183.65	12.4386 ± 0.8725	80.40
CGHN-Net	7.8121	37.6507	274.60	6.8320 ± 0.0089	146.37

**Table 12:** Segmentation performance on the public CFD after three-seed retraining (mean ± standard deviation).

Model	Dice	IoU	Precision	Recall	FP Area Ratio
UNet++	0.6383 ± 0.0126	0.4760 ± 0.0143	0.5072 ± 0.0247	0.8981 ± 0.0217	0.0137 ± 0.0015
BGCrack	0.6380 ± 0.0081	0.4743 ± 0.0100	0.5920 ± 0.0462	0.7439 ± 0.0472	0.0076 ± 0.0020
CGHN-Net	0.6679 ± 0.0162	0.5028 ± 0.0182	0.5177 ± 0.0214	0.9486 ± 0.0085	0.0153 ± 0.0013

**Table 13:** Sequence-aware paired statistical comparison between CGHN-Net and UNet++ over the 87 test images.

Metric	Mean Paired Difference	95% Block-Bootstrap CI	Block Length	Holm-Adjusted *p*	Win Rate (%)
Dice	0.0102	[0.0064, 0.0142]	3	<0.0001	67.8
		[0.0066, 0.0138]	5	<0.0001	
		[0.0076, 0.0130]	10	0.0195	
IoU	0.0154	[0.0100, 0.0213]	3	<0.0001	69.0
		[0.0102, 0.0206]	5	<0.0001	
		[0.0119, 0.0196]	10	0.0195	
Precision	0.0105	[0.0047, 0.0171]	3	0.0014	58.6
		[0.0045, 0.0169]	5	0.0082	
		[0.0050, 0.0167]	10	0.0469	
Recall	0.0092	[0.0030, 0.0153]	3	0.0016	64.4
		[0.0032, 0.0147]	5	0.0082	
		[0.0040, 0.0147]	10	0.0469	
FP area ratio reduction	0.00055	[0.00024, 0.00090]	3	0.0014	56.3
		[0.00023, 0.00091]	5	0.0082	
		[0.00025, 0.00092]	10	0.0469	

**Table 14:** Comparison of raw and post-processed segmentation results for UNet++ and CGHN-Net.

Model	Evaluation Output	Dice	IoU	Precision	Recall	FP Area Ratio
UNet++	Raw thresholded output	0.8540 ± 0.0116	0.7565 ± 0.0183	0.8666 ± 0.0022	0.8661 ± 0.0242	0.0049 ± 0.0001
UNet++	Post-processed output	0.8590 ± 0.0107	0.7649 ± 0.0170	0.8682 ± 0.0041	0.8650 ± 0.0258	0.0047 ± 0.0002
CGHN-Net	Raw thresholded output	0.8650 ± 0.0046	0.7732 ± 0.0088	0.8790 ± 0.0169	0.8752 ± 0.0243	0.0042 ± 0.0009
CGHN-Net	Post-processed output	0.8682 ± 0.0055	0.7791 ± 0.0102	0.8780 ± 0.0161	0.8734 ± 0.0266	0.0042 ± 0.0008

**Table 15:** False-positive evaluation on the held-out crack-free subset from Road Section D.

Model	Raw Image-Level FAR	Post Image-Level FAR	Raw FP Area Ratio	Post FP Area Ratio
B0	0.3700 ± 0.0556	0.3100 ± 0.0529	0.0037 ± 0.0010	0.0035 ± 0.0009
UNet++	0.2400 ± 0.0264	0.1766 ± 0.0351	0.0023 ± 0.0002	0.0021 ± 0.0002
B1	0.4166 ± 0.0808	0.3100 ± 0.0556	0.0040 ± 0.0008	0.0037 ± 0.0007
B1 + OHEM	0.3330 ± 0.0115	0.2833 ± 0.0288	0.0042 ± 0.0008	0.0039 ± 0.0007
CGHN-Net	0.1866 ± 0.0723	0.1300 ± 0.0335	0.0019 ± 0.0006	0.0018 ± 0.0006

**Table 16:** Ablation results for CGSG and HNBS Loss (mean ± standard deviation).

Model	CGSG	HNBS Loss	Dice	IoU	Precision	Recall	FP Area Ratio
B0	×	×	0.8624 ± 0.0063	0.7708 ± 0.0105	0.8624 ± 0.0189	0.8796 ± 0.0280	0.0049 ± 0.0007
B0 + HNBS	×	√	0.8526 ± 0.0033	0.7547 ± 0.0061	0.8723 ± 0.0141	0.8498 ± 0.0189	0.0045 ± 0.0005
B1	√	×	0.8643 ± 0.0021	0.7717 ± 0.0047	0.8670 ± 0.0125	0.8769 ± 0.0172	0.0047 ± 0.0005
CGHN-Net	√	√	0.8682 ± 0.0055	0.7791 ± 0.0102	0.8780 ± 0.0161	0.8734 ± 0.0266	0.0042 ± 0.0008

## Data Availability

The datasets used and/or analyzed during the current study are available from the corresponding author upon reasonable request.
